# Analysis of “Stand Your Ground” Self-defense Laws and Statewide Rates of Homicides and Firearm Homicides

**DOI:** 10.1001/jamanetworkopen.2022.0077

**Published:** 2022-02-21

**Authors:** Michelle Degli Esposti, Douglas J. Wiebe, Antonio Gasparrini, David K. Humphreys

**Affiliations:** 1 Department of Social Policy and Intervention, University of Oxford, Oxford, United Kingdom; 2 Department of Biostatistics and Epidemiology, University of Pennsylvania, Philadelphia; 3 Department of Public Health, Environments and Society, London School of Hygiene & Tropical Medicine, London, United Kingdom; 4 Centre for Statistical Methodology, London School of Hygiene & Tropical Medicine, London, United Kingdom

## Abstract

**Importance:**

Most US states have amended self-defense laws to enhance legal immunities for individuals using deadly force in public. Despite concerns that “stand your ground” (SYG) laws unnecessarily encourage the use of deadly violence, their impact on violent deaths and how this varies across states and demographic groups remains unclear.

**Objective:**

To evaluate the association of SYG laws with homicide and firearm homicide, nationally and by state, while considering variation by the race, age, and sex of individuals who died by homicide.

**Design, Setting, and Participants:**

This cohort study used a controlled, multiple-baseline and -location interrupted time series design, using natural variation in the timings and locations of SYG laws to assess associations. Changes in homicide and firearm homicide were modeled using Poisson regression analyses within a generalized additive model framework. Analyses included all US states that enacted SYG laws between 2000 and 2016 and states that did not have SYG laws enacted during the full study period, 1999 to 2017. Data were analyzed from November 2019 to December 2020.

**Exposures:**

SYG self-defense laws enacted by statute between January 1, 2000, to December 31, 2016.

**Main Outcomes and Measures:**

The main outcomes were statewide monthly rates of homicide and firearm-related homicide (per 100 000 persons) from January 1, 1999, to December 31, 2017, grouped by characteristics (ie, race, age, sex) of individuals who died by homicide.

**Results:**

Forty-one states were analyzed, including 23 states that enacted SYG laws during the study period and 18 states that did not have SYG laws, with 248 358 homicides (43.7% individuals aged 20-34 years; 77.9% men and 22.1% women), including 170 659 firearm homicides. SYG laws were associated with a mean national increase of 7.8% in monthly homicide rates (incidence rate ratio [IRR],1.08; 95% CI, 1.04-1.12; *P* < .001) and 8.0% in monthly firearm homicide rates (IRR, 1.08; 95% CI, 1.03-1.13; *P* = .002). SYG laws were not associated with changes in the negative controls of suicide (IRR, 0.99; 95% CI, 0.98-1.01) or firearm suicide (IRR, 1.00; 95% CI, 0.98-1.02). Increases in violent deaths varied across states, with the largest increases (16.2% to 33.5%) clustering in the South (eg, Alabama, Florida, Georgia, Louisiana). There were no differential associations of SYG laws by demographic group.

**Conclusions and Relevance:**

These findings suggest that adoption of SYG laws across the US was associated with increases in violent deaths, deaths that could potentially have been avoided.

## Key Points

**Question:**

Are “stand your ground” (SYG) laws associated with increases in violent deaths, and does this vary by US state?

**Findings:**

In this cohort study assessing 41 US states, SYG laws were associated with an 8% to 11% national increase in monthly rates of homicide and firearm homicide. State-level increases in homicide and firearm homicide rates reached 10% or higher for many Southern states, including Alabama, Florida, Georgia, and Louisiana.

**Meaning:**

These findings suggest that SYG laws were associated with increased homicides each year and that the laws should be reconsidered to prevent unnecessary violent deaths.

## Teaser

This cohort study evaluates the association of “stand your ground” laws with US homicide and firearm rates nationally and by state.

## Introduction

Every year, more than 19 000 people in the United States die from homicide, and most of these violent deaths are attributable to injuries sustained from firearms. Rates of homicide and firearm homicide are markedly higher in the US than any other high-income county.^1,2,3^ In 2020, the US saw a further upsurge, with homicide rates increasing by approximately 30%.^4^ These deaths are preventable; yet there is limited evidence on how current legislation and policy may not only be failing to prevent harm but may also be contributing to it.^5^

“Stand your ground” (SYG) laws, also known as *shoot first* laws, overwrite the common law principle of a “duty to retreat,” creating the possibility for individuals to use deadly force in self-defense in public as a first, rather than last, resort (eMethods in the Supplement).^6^ Florida was the first state to enact an SYG law by statute in 2005, and then 23 states enacted SYG laws soon after, between 2006 and 2008. Although the uptake was initially concentrated in the South, by 2021, thirty states had enacted SYG laws, and this number continues to increase as a raft of ongoing bills make their way through state legislatures.^7^

Advocates claim that SYG laws enhance public safety by deterring predatory crime through an increased threat of retaliatory violence.^8^ Critics, on the other hand, argue that the laws are unnecessary, and may threaten public safety by emboldening the use of deadly violence in public encounters in which violence and injury that could have safely been avoided.^9^ There are also concerns that the laws exacerbate social inequalities in experiencing violent crime, since implicit and explicit biases of threat perception discriminate against and cause disproportionate harms among minority groups, such as Black people.^10,11,12^ Anecdotally, critics’ concerns have been realized in an increasing number of shootings of young Black men (eg, Trayvon Martin, Jordan Davis, and Markeis McGlockton) where self-defense has been claimed.^12^ These high-profile incidents underline the controversy surrounding SYG laws and have served to galvanize the Black Lives Matter movement.^13^

Although previous studies have associated SYG laws with increases in homicide,^14,15,16,17^ a 2021 systematic review highlighted key weaknesses of the current evidence.^18^ First, the national impacts of SYG laws are undetermined because there is a lack of studies with robust study designs.^18^ Second, very few studies examine the differential impacts of SYG laws on demographic groups, including racial or ethnic minority groups. Third, there is a marked discrepancy between US-wide analyses and single-state analyses. US-wide analyses report either no associations or small increases in homicide associated with enacting SYG laws^15,18,19,20^; whereas single-state analyses, which almost exclusively evaluated Florida’s SYG law, identify substantial increases in homicide (24% to 27%).^14,21,22^ The generalizability of the evidence beyond Florida remains unclear.

Since the laws continue to be adopted across the US, with 2 states passing SYG bills in early 2021 and 14 states currently having SYG bills under active consideration,^7^ it is crucial to advance the evidence on SYG laws across the US and whether the laws show differential associations by state and demographic group. Our study aims to fill this gap. We obtained restricted access mortality data by special request to the Centers for Disease Control and Prevention (CDC) that includes all medical records on causes of death from January 1, 1999, to December 31, 2017. We used an interrupted time-series (ITS) design that exploited temporal and state variation in the enactment of the laws to strengthen the evidence on the outcomes associated with enacting SYG laws across the US.

## Methods

Ethical approval was waived by Departmental Research Ethics Committee (DREC), University of Oxford, because the study uses fully anonymized administrative data previously collected by government bodies and official sources. The data cannot be traced back to an individual. We followed relevant recommendations set out in the Guidelines for Reporting Evaluations based on Observational Methodology (GREOM) and Strengthening the Reporting of Observational Studies in Epidemiology (STROBE) reporting guideline. The study protocol and statistical analysis plan (https://osf.io/s6xp4/) and analytical code (https://osf.io/vmx2y/) have been made freely available at the Open Science Framework.

### Study Design

We used a multiple-baseline and -location ITS design to estimate the association of SYG laws with changes in homicide and firearm homicide rates across the US, both at the national and state levels. The staggered adoption of SYG laws across states offers a unique opportunity to reduce confounding in ITS (pre-post) evaluations by including states that did not enact SYG laws in the control group.^23,24^ Observed and unobserved state- and time-varying confounding is therefore minimized, as the same confounding would have to present at all 23 different timings in each of the 23 states that enacted SYG laws while not occurring in any of the 18 states that did not have SYG laws enacted during the study period.

### Intervention

We defined an SYG law as a legislative statute that extended the legal right to use lethal force in self-defense to anywhere the individual has the right to be (ie, public places) (eMethods in the Supplement). We systematically investigated self-defense laws in all 50 US states and classified each state by their variant of self-defense law (eMethods, eTable 1, and eFigure 1 in the Supplement). States were included in the analysis if they were classified as an SYG state, enacting SYG laws between January 1, 2000, and December 31, 2016. A reduced period of 2000 to 2016 was used to ensure that at least 12 months in the preintervention and postintervention period for modeling purposes. States were also included if they could serve as a comparison non-SYG state by not having an SYG law enacted by statute or upholding SYG law principles through case law, throughout the study period. We included 41 states in the analyses: 23 SYG states and 18 non-SYG states (eTable 2 in the Supplement). We excluded the remaining 9 states from the analyses because 2 states (Utah and Iowa) lacked sufficient data during the study period to model trends and 7 states upheld principles of SYG law by case law even if not encoded in a statute. Including these states in the analysis would dilute the contrast between SYG and non-SYG states and bias estimates owing to intervention contamination effects.^25^

### Outcomes

We modeled time series data for state-level monthly counts of homicide (primary outcome) and firearm homicide (secondary outcome) between January 1999 to December 2017 (eMethods, eFigure 2, eFigure 3, and eTable 3 in the Supplement). Outcome data were obtained by special request from the CDC’s Restricted Use Vital Statistics, which provided microdata on Multiple Cause of Death based on coroner determinations of cause of death for more than 99% of all deaths in the US. We further stratified outcomes by race (White vs Black and other races), age group (0-19, 20-34, and ≥35 years), and sex (male and female). Race was determined by medical death certificates in accordance with standards set forth by the Office of Management and Budget. Other races includes all races other than White or Black under the main categories of American Indian, Asian, and Pacific Islander. We stratified outcomes by race to determine whether the SYG laws had differential assoications by population subgroup, including by race. We identified and excluded any outliers from 1-event mass death events (eg, the September 11, 2001, terrorist attack) from the analyses (eMethods and eTable 4 in the Supplement).

We used negative control outcomes of suicide and firearm suicide to check for time-varying confounding.^26^ We selected these outcomes because we hypothesized that they would be similarly affected by changes over time that might confound the association between SYG law enactment and changes in homicide and firearm homicide but would not be affected by the intervention itself. Such time-varying confounders included economic shifts (eg, an economic recession), changes in recording practices, and changes in firearm regulation and availability.

### Statistical Analysis

We ran Poisson regression analyses within a generalized additive model (GAM) framework to estimate the association of SYG laws with homicide and firearm homicide. Outcomes were modeled as a quasi-Poisson distribution owing to evidence of overdispersion. State population sizes by state and year, disaggregated by race, age group, and sex as relevant, were used as an offset variable to model rates per 100 000 persons directly. Our main exposure was a dummy variable indicating the presence of SYG law across groups (SYG vs non-SYG states) and over time (before and after SYG laws were enacted in SYG states). The dummy variable thus reflects a group-by-time interaction: 0 for non-SYG states and for the pre-enactment period in SYG laws and 1 for the postenactment period in SYG states. GAMs, as opposed to generalized linear mixed models (GLMMs), were used because there was significant evidence that long-term trends violated assumptions of linearity (eAppendix, eFigure 4, and eTable 5 in the Supplement). GAMs can more flexibly smooth nonlinear trends while penalizing overfitting through generalized cross-validation.^27^ To check the robustness of this penalization approach, we present estimates from nonpenalized GLMMs in the eAppendix in the Supplement.

Informed by a previous systematic review,^18^ we hypothesized that the outcomes associated with SYG laws would follow a step-change pattern (ie, abrupt sustained change in level). The adoption of SYG laws was therefore estimated as a fixed effect, comparing differences in rates before and after SYG laws were enacted in the 23 SYG states, as well as with the 18 non-SYG states, while adjusting for seasonality and state-specific long-term trends. Both seasonality and long-term trends were modeled independently for each outcome. Seasonality was modeled using harmonics (Fourier series of pairs of sine and cosine functions) to smooth smaller repeating patterns in a year (ie, seasons).^24,28^ State-specific trends were independently smoothed by specifying a factor-by-curve interaction to allow for differences in long-term trends between states.^29^ The smoothness of trends was constrained to be equal across states to prioritize model parsimony and because there was no theoretical reason to assume that states should be smoothed to a differential degree. The equation for the base model is outlined in the eMethods in the Supplement. Results can be interpreted as the relative risk of monthly homicides or firearm homicides in the presence vs absence of SYG laws (ie, incidence rate ratio [IRR]). Model fit was checked through an analysis of the residuals, including data visualizations and inspection of the distribution of autocorrelations.

Stratified analyses were conducted to investigate whether associations of SYG laws with violent deaths differed by race, age group, and sex. Formal tests assessed whether the estimates varied across demographic groups. Specifically, approximate Wald tests (also known as Z*-tests*) were used to compare stratified model estimates and test for differences within each demographic group (eg, White vs Black and other races).^30^ These model comparisons were used to assess whether SYG laws were differentially associated with violent deaths by subgroup.

We also investigated state-by-state differences in estimates of the enactment of SYG laws by fitting separate quasi-Poisson regression models as interrupted times series analyses for each SYG state. Here, the main exposure is a dummy variable coding the absence (0) and presence (1) of SYG laws within SYG states alone, and thus implements a segmented regression analysis.^24,31^ In these ITS models, we smoothed nonlinearity in long-term trends in 2 ways: fitted cubic terms for long-term trends and restricted the period to 3 years (ie, 36 months) before and after SYG laws were enacted, and we assumed fitted linear long-term trends (eAppendix in the Supplement). State-level ITS models estimates were then pooled in fixed-effects meta-analyses. All analyses were conducted in R statistical software version 3.6.3 (R Project for Statistical Computing); the multiple location and baseline ITS GAMs were fitted using the R package *mgcv* while the simple ITS models were pooled using *metafor*.^32,33^ *P* values were 2-sided, and statistical significance was set at *P* < .05. Data were analyzed from November 2019 to December 2020.

## Results

**Figure 1.**
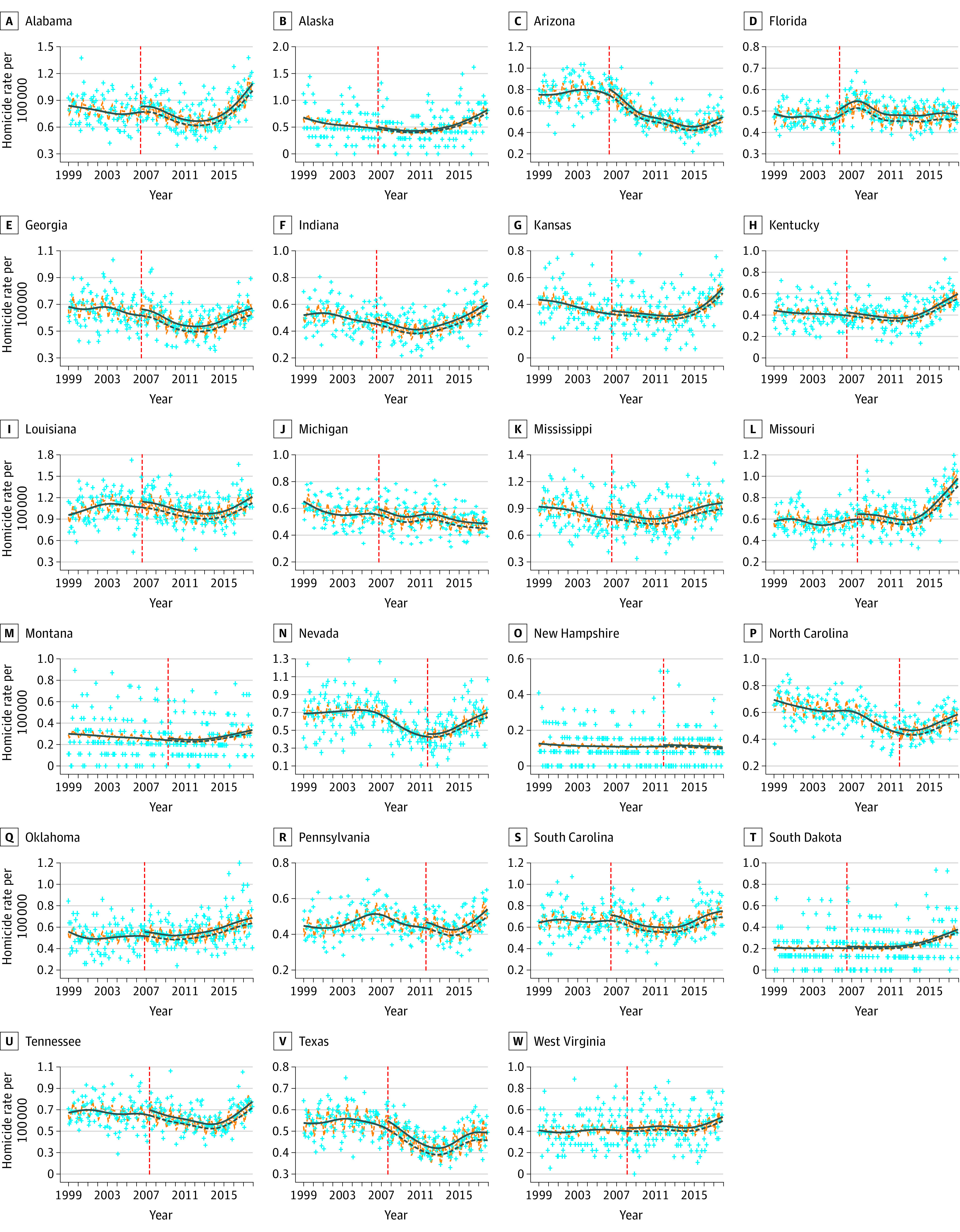
Associations of “Stand Your Ground” Laws With Changes in Monthly Homicide Rates Across the United States Crosses indicate monthly homicide rates; blue solid lines, smoothed trends for observed rates; orange dotted lines, smoothed seasonality for observed rates; red dotted lines represent the enactment of SYG laws; and blue dotted lines, the smoothed trend for the counterfactual (ie, the expected trend in the absence of an SYG law).

The analysis included 41 states with 248 358 homicides (43.7% individuals aged 20-34 years; 77.9% men and 22.1% women), including 184 495 homicides in 23 SYG states and 63 863 homicides in 18 non-SYG states, and 170 659 firearm homicides, including 129 831 firearm homicides in 23 SYG states and 40 828 firearm homicides in 18 non-SYG states). The Table and eTable 6 in the Supplement present monthly rates and counts during the study period. Between 1999 and 2017, trends in monthly homicide and firearm homicide rates varied between states and did not follow simple linear trends. While there were gradual declines between 1999 and 2014, approximately half of all states experienced an uptick in homicide rates in recent years, irrespective of whether states had or had not enacted SYG laws (Figure 1; eFigure 5 and eFigure 6 in the Supplement). The negative control outcomes of suicide and firearm suicides rates mostly showed increasing trends from 1999 to 2017 (eFigure 7 and eFigure 8 in the Supplement). Between 1999 and 2017, homicide and suicide rates were higher in states with SYG laws (mean [SD]: 0.55 [0.25] homicides per 100 000 persons and 1.24 [0.38] suicides per 100 000 persons) compared with non-SYG states (mean [SD]: 0.31 [0.22] homicides per 100 000 persons and 1.03 [0.45] suicides per 100 000 persons) (Table). Within SYG states and compared with the period before SYG laws were enacted, after SYG laws were enacted there were higher rates of homicide (mean [SD], 0.54 [0.26] vs 0.55 [0.25] homicides per 100 000 population) and suicide (mean [SD], 1.13 [0.35] vs 1.32 [0.38] suicides per 100 000 population).

**Table.** Associations of Stand Your Ground (SYG) Laws With Changes in Homicide and Firearm Homicide Rates Across the United States

Characteristic	Monthly rates per 100 000 population, mean (SD)				Step change, IRR (95% CI)	Between-group *P* value^a^
	SYG states (n = 23)			Non-SYG states, 1999-2017 (n = 18)		
	1999-2017	Before law	After law			
**Homicide**						
Overall	0.55 (0.25)	0.54 (0.26)	0.55 (0.25)	0.31 (0.22)	1.08 (1.04-1.12)^b^	NA
Race						
White	0.32 (0.17)	0.33 (0.18)	0.31 (0.16)	0.18 (0.14)	1.10 (1.05-1.15)^b^	.25
Black and other^c^	1.45 (0.87)	1.45 (0.89)	1.46 (0.85)	0.96 (1.04)	1.05 (1.00-1.11)^d^	
Age, y						
0-19	0.32 (0.26)	0.32 (0.26)	0.32 (0.26)	0.27 (0.46)	1.08 (0.99-1.17)	NA
20-34	1.12 (0.65)	1.10 (0.65)	1.14 (0.65)	0.66 (0.61)	1.08 (1.02-1.13)^e^	.94
≥35	0.46 (0.24)	0.45 (0.25)	0.46 (0.24)	0.24 (0.21)	1.07 (1.02-1.12)^e^	.82
Sex						
Male	0.85 (0.43)	0.83 (0.43)	0.87 (0.44)	0.48 (0.38)	1.08 (1.04-1.13)^b^	.10
Female	0.25 (0.16)	0.26 (0.16)	0.24 (0.15)	0.15 (0.15)	1.02 (0.97-1.08)	
**Firearm homicide**						
Overall	0.38 (0.21)	0.36 (0.20)	0.39 (0.22)	0.19 (0.17)	1.08 (1.03-1.13)^e^	NA
Race						
White	0.20 (0.13)	0.20 (0.14)	0.20 (0.13)	0.09 (0.10)	1.10 (1.04-1.17)^e^	.20
Black and other	1.08 (0.77)	1.03 (0.76)	1.12 (0.78)	0.65 (0.78)	1.04 (0.97-1.11)	
Age, y						
0-19	0.19 (0.19)	0.19 (0.19)	0.20 (0.20)	0.14 (0.31)	1.07 (0.96-1.19)	NA
20-34	0.90 (0.59)	0.85 (0.57)	0.94 (0.60)	0.50 (0.53)	1.04 (0.97-1.12)	.73
≥35	0.28 (0.18)	0.26 (0.18)	0.29 (0.19)	0.12 (0.15)	1.07 (1.01-1.14)^d^	.93
Sex						
Male	0.63 (0.38)	0.59 (0.36)	0.66 (0.39)	0.33 (0.31)	1.07 (1.01-1.12)^d^	.70
Female	0.13 (0.11)	0.13 (0.11)	0.14 (0.11)	0.06 (0.10)	1.08 (1.01-1.17)^d^	
Controls						
Suicide	1.24 (0.38)	1.13 (0.35)	1.32 (0.38)	1.03 (0.45)	0.99 (0.98-1.01)	NA
Firearm suicide	0.74 (0.27)	0.69 (0.25)	0.78 (0.27)	0.50 (0.36)	1.00 (0.98-1.02)	NA

Abbreviations: IRR, incidence rate ratios; NA, not applicable (reference group for comparison within sociodemographic groups); SYG, stand your ground.

^a^ *P* values are based on Wald tests comparing stratified models within each sociodemographic group.

^b^ *P* < .001.

^c^ Other races includes all races other than White or Black under the main categories of American Indian, Asian, and Pacific Islander.

^d^ *P* < .05.

^e^ *P* < .01.

The enactment of SYG laws was associated with a mean national increase of 7.8% in monthly homicide rates (IRR, 1.08; 95% CI, 1.04-1.12; *P* < .001) and 8.0% in monthly firearm homicide rates (IRR, 1.08; 95% CI, 1.03-1.13; *P* = .002) (Table and Figure 1; eFigure 6 in the Supplement). SYG laws were not associated with changes in the 2 negative control outcomes: suicide (IRR, 0.99; 95% CI, 0.98-1.01; *P* = .58) or firearm suicide rates (IRR, 1.00; 95% CI, 0.98-1.02; *P* = .94) (eFigure 7 and eFigure 8 in the Supplement). Separate ITS analyses for SYG states only, pooled via meta-analyses, estimated higher increases of 9.9% for homicide (IRR, 1.10; 95% CI, 1.07-1.13; *P* < .001) and 10.8% for firearm homicide (IRR, 1.11; 95% CI, 1.07-1.15; *P* < .001). No changes were observed for the negative control suicide outcomes.

The penalization approach was tested by fitting a series of nonpenalized GLMMs using polynomials to model nonlinear trends at the national and state level (eAppendix in the Supplement). Results from the GLMMs replicated our main findings, with associated increases of 9.5% for homicide (IRR, 1.09; 95% CI, 1.07-1.12; *P* < .001) and 9.1% for firearm homicide (IRR = 1.09; 95% CI, 1.06-1.12; *P* < .001) (eTable 7 in the Supplement). Nonlinear models were further supplemented by restricting the study period to a smaller temporal window (ie, 36 months before and after the enactment of SYG laws) and fitting state-specific ITS models with linear trends (eAppendix in the Supplement). Consistently, these linear models identified an 8.9% increase for homicide (IRR, 1.09; 95% CI, 1.05-1.13; *P* < .001) and a 9.2% increase for firearm homicide (IRR, 1.09; 95% CI, 1.04-1.14; *P* < .001) following the enactment of SYG laws (eFigure 9 and eFigure 10 in the Supplement). There continued to be no association between SYG laws and suicide (IRR, 0.99; 95% CI, 0.97-1.02; *P* = .46) or firearm suicide (IRR, 0.99; 95% CI, 0.96-1.02; *P* = .47).

**Figure 2.**
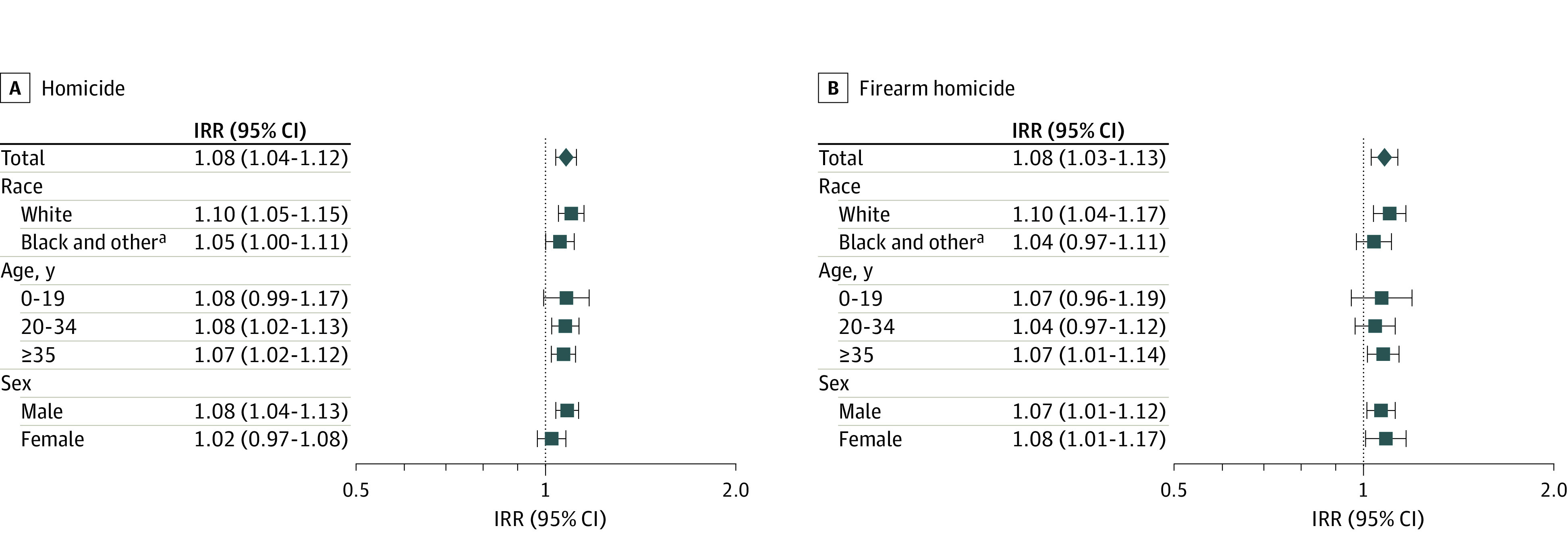
Associations of Stand Your Ground Laws With Changes in Monthly Homicide and Firearm Homicide Rates by Race, Age Groups, and Sex IRR indicates incidence rate ratio, given as rate per 100 000 population.

No statistically significant differences by race, age group, or sex of individuals who died by homicide were identified (Table; eTable 8 in the Supplement). However, stratified models showed more pronounced increases in some demographic groups (Figure 2). The largest increases were seen for White individuals (IRR, 1.10; 95% CI, 1.05-1.15; *P* < .001) and for males (IRR, 1.08; 95% CI, 1.04-1.13; *P* < .001). There was inconsistent evidence that the enactment of SYG laws was associated with increases for persons aged 0 to 19 years or females. The main models (GAMs) identified no significant associations, whereas supplementary models (GLMMs) estimated significant associations (eTable 7 in the Supplement). This is likely because the subgroups of individuals aged 0 to 19 years and females had the smallest number of individuals who died by homicide, thus model estimation suffered from low counts and increased uncertainty (eTable 6 in the Supplement).

**Figure 3.**
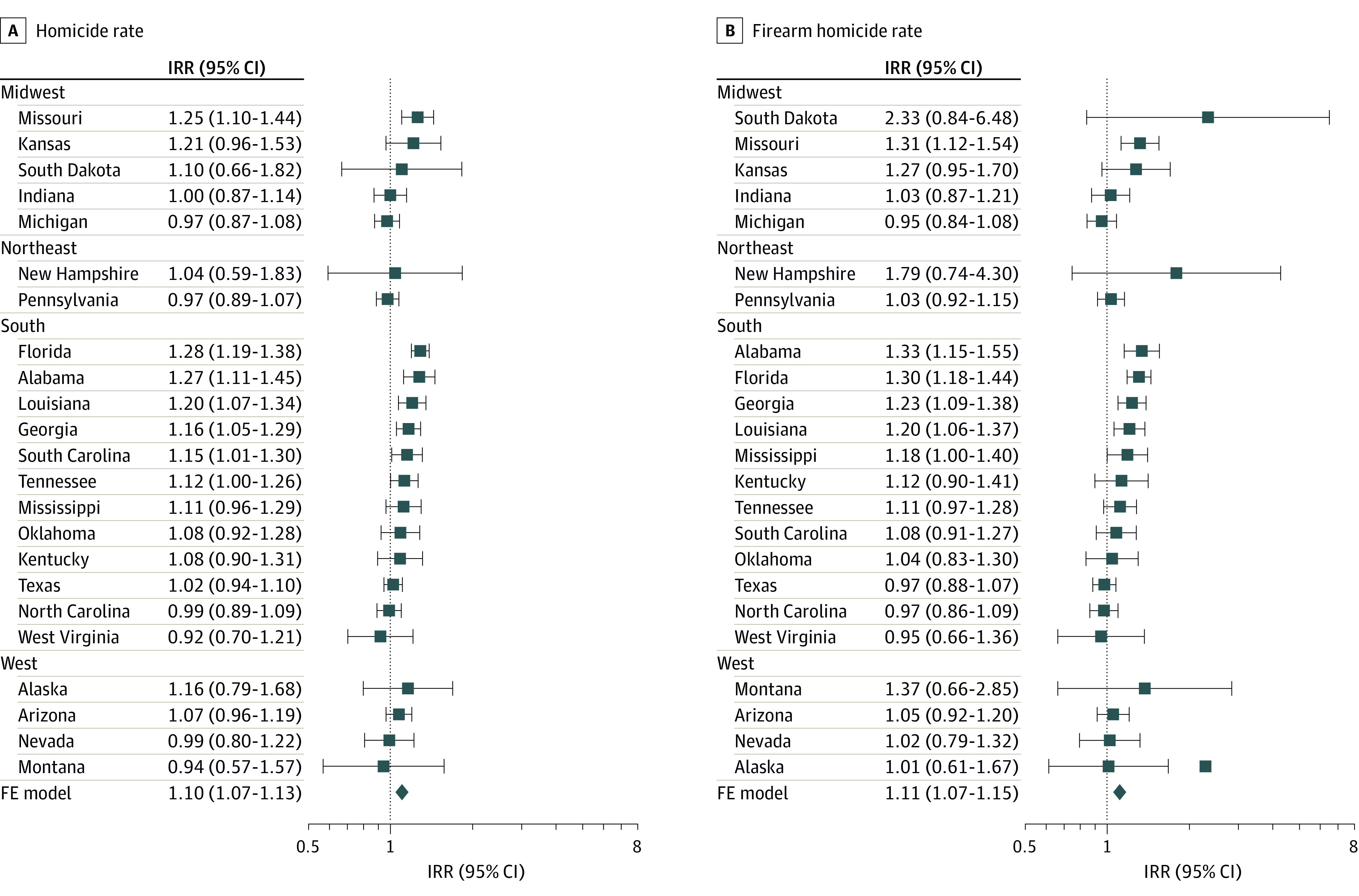
Forest Plot of State and National Associations of Stand Your Ground (SYG) Laws With Homicide and Firearm Homicide Estimated by Separate Interrupted Time Series Models With Nonlinear Trends for Each SYG State Note each plot is grouped by US region (Midwest, Northeast, South, West) and ordered by estimated effect size. FE indicates fixed-effects.

Associations of SYG laws with violent deaths differed by state (Figure 3). Large increases for homicide and firearm homicide rates were associated with the enactment of SYG laws in Alabama, Florida, Georgia, Louisiana, and Missouri. These increases ranged from 16.2% to 33.5%, with firearm homicides typically showing larger increases than total homicides. SYG laws were not significantly associated with changes in homicides or firearm homicides rates in a handful of states, including Arizona, Indiana, Michigan, Nevada, Oklahoma, Texas, and West Virginia. We explored between-state heterogeneity by visualizing state-specific associations across the US, which showed that larger associations clustered in Southern states (Figure 3; eFigure 11 in the Supplement).

## Discussion

This cohort study found that the enactment of SYG laws was associated with an abrupt and sustained 8% to 11% national increase in monthly homicide and firearm homicide rates, contributing an extra 58 to 72 homicides each month. This monthly increase alone exceeds total rates of homicides in most Northern and Western European countries today.^34^ Estimates are consistent with previous studies^15,17^; but this study advances the evidence by examining SYG laws over an extended timeframe and using enhanced mortality data to investigate differential associations by state and demographic group.

Although the enactment of SYG laws was not associated with significant change in violent deaths in all states, there was no evidence that SYG laws were associated with decreases in homicide or firearm homicide. Our analyses show that increases in homicide associated with SYG laws were not restricted to Florida.^14,21,22^ Comparable changes were observed in at least 8 other states at different times across the US, although the largest increases clustered in the South and in states that were early adopters of SYG laws (2005-2007). The association between SYG laws and increases in violent deaths cannot be attributed to distortion by a single state outlier (eg, Florida),^18^ but may be attributed to time-varying confounders specific to the South and/or the early enactment of SYG laws. The between-state heterogeneity suggests that SYG laws alone may not be sufficient in explaining increases in homicide. Understanding the factors shaping these differential associations between states, such as regions endorsing the use of self-protective violence,^35^ existing state firearm legislation, and firearm availability, is key to understanding how and why legally expanding the right to use deadly violence in public is associated with increases in homicides in some states but not others.

Despite concerns that SYG laws exacerbate social inequalities in experiencing violent crime,^13^ we did not find differential associations by demographic group. SYG laws were associated with mean increases in homicide and firearm homicide rates irrespective of race, age group, or sex of individuals who died by homicide .^18^ At least in terms of homicide, these findings do not lend support for the claim that SYG laws widen racial disparities. However, our analyses are based on causes of death and thus can only examine homicide. We do not examine nonfatal injury, patterns of racial concordance between the deceased and the defendant, or patterns in legal outcomes (eg, conviction or acquittal rates) following enactment of SYG laws. Owing to the ongoing context of racism in the US^10,11,12^ and previous studies showing multiple pathways through which SYG laws may exacerbate social injustice (eg, via racialized threat perceptions),^36,37^ future research should aim to assess the disproportional impacts and implications of SYG laws for disadvantaged groups, such as Black individuals.

### Limitations

This study has some limitations. Our study period was limited to ending in December 2017 because this was the most recent available data at the time of conducting the research. Five additional states have since enacted SYG laws, which we were unable to evaluate as they fell outside our study period. Iowa was also excluded from the analyses because its SYG law was enacted in July 2017, so it had insufficient time points to model the postintervention period. Most SYG states included in this study were early adopters of SYG law, with 74% adopting the law between October 2005 and September 2007. This limited variation in the enactment timing reduces the generalizability of our findings to more recent adoptions of SYG laws. Low homicide counts among demographic groups, especially for persons aged 0 to 19 years and females, restricted modeling power and certainty for estimating associations for these subgroups. Nevertheless, these findings echoed the limited number of analyses that have previously investigated distributional outcomes associated with SYG laws.^18^ Although the design and advanced statistical modeling used here minimize confounding, it is still possible that the unobserved heterogeneity across states and changes that occurred around the same time of each SYG enactment mean our estimates reflect the outcomes associated with SYG laws plus a spurious effect from confounding. The most likely source of confounding is that the as-if-random assumption of the timings of SYG law enactment across states was violated owing to another factor (eg, high-profile self-defense cases, changes in societal attitudes, coordinated campaigns by lobbyists),^38^ causing both the enactment of SYG law and increases in violent deaths in the state.^39^ However, in the absence of a feasible randomized clinical trial, we present strong alternative evidence by controlling for observed and unobserved confounding by design and including a series of robustness checks.

## Conclusions

This cohort study found that the staggered adoption of SYG laws in US states was associated with increases in homicide and firearm homicide rates across the US. These increases reach 10% and higher in several Southern states, while no states had significant reductions in violent deaths, as advocates often argue when justifying these laws. The accumulation of evidence established in this and other studies point to harmful outcomes associated with SYG laws. Despite this, SYG laws have now been enacted in most states, and the uptake of new SYG bills continues to be popular, unnecessarily risking lives.

## Notes

Supplement. **eMethods.** Stand Your Ground and Self-Defense Laws by State **eAppendix.** Evidence of Nonlinear Trends, Nonpenalized Approach, and Restricted Linear Analyses **eFigure 1.** Visualization of the Multiple Baselines Using Staggering of the Enactment of SYG Laws and Multiple Locations Using 23 SYG States During the Study Period, 1999 to 2017 **eFigure 2.** Data Visualization Showing the Suspected Coding Error in the Data for Race in Texas From September 2007 to February 2009 **eFigure 3.** Plots Showing Imputed Data by Bounded Random Sampling to Correct for the Suspected Coding Error in the Data for Race in Texas From September 2007 to February 2009 **eFigure 4.** Plots of Partial Autocorrelation Functions for GLMM With Linear Trends and GAMs for Homicide and Firearm Homicide **eFigure 5.** Estimated Associations of SYG Laws With Monthly Homicide Rates Across the US **eFigure 6.** Estimated Associations of SYG Laws With Monthly Firearm Homicide Rates Across the US **eFigure 7.** Estimated Associations of SYG Laws With Monthly Suicide Rates Across the US **eFigure 8.** Estimated Associations of SYG Laws With Monthly Firearm Suicide Rates Across the US **eFigure 9.** Estimated Associations of SYG Laws With Monthly Homicide and Firearm Homicide Rates in SYG States by Restricted ITS Models With Linear Trends **eFigure 10.** Forest Plot of Fixed-Effects Meta-analysis Pooling Restricted ITS Models With Linear Trends for the Association of SYG Laws With Homicide and Firearm Homicide Rates in SYG States **eFigure 11.** State-Specific Associations of SYG Laws With Monthly Homicide Rates Estimated by Separate ITS Models With Nonlinear Trends for Each SYG State **eTable 1.** Self-defense Laws Across All 50 US States (Excluding District of Columbia) **eTable 2.** Intervention SYG States, Comparison Non-SYG States, and Excluded States **eTable 3.** Outcomes and Their Corresponding Selected Cause of Death Group and *ICD-10* Details **eTable 4.** Data Outliers Caused by 1-Off Events **eTable 5.** AIC Values for Different Combinations of Polynomials for Modelling State and National Trends in GLMMs for Homicides **eTable 6.** Monthly Counts of Homicide, Firearm Homicide, Suicide, and Firearm Suicide in the Absence and Presence of SYG Laws **eTable 7.** Estimated Associations of SYG Laws With Homicide and Firearm Homicide Rates Across the US Using Nonpenalized GLMMs With Polynomials **eTable 8.** Estimated Association of SYG Laws With Homicide and Firearm Homicide Rates by Race Across the US Using Data With Suspected Errors
eReferences
