# Error in Figure 3

**DOI:** 10.1001/jamanetworkopen.2022.9270

**Published:** 2022-04-06

In the Original Investigation titled “Analysis of ‘Stand Your Ground’ Self-defense Laws and Statewide Rates of Homicides and Firearm Homicides,”^1^ published February 21, 2022, there was an extraneous box in Figure 3. This article has been corrected.^1^
