# Supplementary material for: Analysis of “Stand Your Ground” Self-defense Laws and Statewide Rates of Homicides and Firearm Homicides
Source: JAMA Netw Open. 2022 Feb 21;5(2):e220077. doi: 10.1001/jamanetworkopen.2022.0077 (PMC8861849; doi:10.1001/jamanetworkopen.2022.0077)
Supplement: Supplement. — eMethods. Stand Your Ground and Self-Defense Laws by State eAppendix. Evidence of Nonlinear Trends, Nonpenalized Approach, and Restricted Linear Analyses eFigure 1. Visualization of the Multiple Baselines Using Staggering of the Enactment of SYG Laws and Multiple Locations Using 23 SYG States During the Study Period, 1999 to 2017 eFigure 2. Data Visualization Showing the Suspected Coding Error in the Data for Race in Texas From September 2007 to February 2009 eFigure 3. Plots Showing Imputed Data by Bounded Random Sampling to Correct for the Suspected Coding Error in the Data for Race in Texas From September 2007 to February 2009 eFigure 4. Plots of Partial Autocorrelation Functions for GLMM With Linear Trends and GAMs for Homicide and Firearm Homicide eFigure 5. Estimated Associations of SYG Laws With Monthly Homicide Rates Across the US eFigure 6. Estimated Associations of SYG Laws With Monthly Firearm Homicide Rates Across the US eFigure 7. Estimated Associations of SYG Laws With Monthly Suicide Rates Across the US eFigure 8. Estimated Associations of SYG Laws With Monthly Firearm Suicide Rates Across the US eFigure 9. Estimated Associations of SYG Laws With Monthly Homicide and Firearm Homicide Rates in SYG States by Restricted ITS Models With Linear Trends eFigure 10. Forest Plot of Fixed-Effects Meta-analysis Pooling Restricted ITS Models With Linear Trends for the Association of SYG Laws With Homicide and Firearm Homicide Rates in SYG States eFigure 11. State-Specific Associations of SYG Laws With Monthly Homicide Rates Estimated by Separate ITS Models With Nonlinear Trends for Each SYG State eTable 1. Self-defense Laws Across All 50 US States (Excluding District of Columbia) eTable 2. Intervention SYG States, Comparison Non-SYG States, and Excluded States eTable 3. Outcomes and Their Corresponding Selected Cause of Death Group and ICD-10 Details eTable 4. Data Outliers Caused by 1-Off Events eTable 5. AIC Values for Different Combinations of Polynomials for [file jamanetwopen-e220077-s001.pdf]

Supplemental Online Content

Degli Esposti M, Wiebe DJ, Gasparrini A, Humphreys DK. Analysis of "stand your ground" self-defense laws and statewide rates of homicides and firearm homicides. *JAMA Netw Open*. 2022;5(2):e220077. doi:10.1001/jamanetworkopen.2022.0077

eMethods. Stand Your Ground and Self-Defense Laws by State
eAppendix. Evidence of Nonlinear Trends, Nonpenalized Approach, and Restricted Linear Analyses
eFigure 1. Visualization of the Multiple Baselines Using Staggering of the Enactment of SYG Laws and Multiple Locations Using 23 SYG States During the Study Period, 1999 to 2017
eFigure 2. Data Visualization Showing the Suspected Coding Error in the Data for Race in Texas From September 2007 to February 2009
eFigure 3. Plots Showing Imputed Data by Bounded Random Sampling to Correct for the Suspected Coding Error in the Data for Race in Texas From September 2007 to February 2009
eFigure 4. Plots of Partial Autocorrelation Functions for GLMM With Linear Trends and GAMs for Homicide and Firearm Homicide
eFigure 5. Estimated Associations of SYG Laws With Monthly Homicide Rates Across the US
eFigure 6. Estimated Associations of SYG Laws With Monthly Firearm Homicide Rates Across the US
eFigure 7. Estimated Associations of SYG Laws With Monthly Suicide Rates Across the US
eFigure 8. Estimated Associations of SYG Laws With Monthly Firearm Suicide Rates Across the US
eFigure 9. Estimated Associations of SYG Laws With Monthly Homicide and Firearm Homicide Rates in SYG States by Restricted ITS Models With Linear Trends
eFigure 10. Forest Plot of Fixed-Effects Meta-analysis Pooling Restricted ITS Models With Linear Trends for the Association of SYG Laws With Homicide and Firearm Homicide Rates in SYG States
eFigure 11. State-Specific Associations of SYG Laws With Monthly Homicide Rates Estimated by Separate ITS Models With Nonlinear Trends for Each SYG State
eTable 1. Self-defense Laws Across All 50 US States (Excluding District of Columbia)
eTable 2. Intervention SYG States, Comparison Non-SYG States, and Excluded States
eTable 3. Outcomes and Their Corresponding Selected Cause of Death Group and *ICD-10* Details
eTable 4. Data Outliers Caused by 1-Off Events
eTable 5. AIC Values for Different Combinations of Polynomials for Modelling State and National Trends in GLMMs for Homicides

eTable 6. Monthly Counts of Homicide, Firearm Homicide, Suicide, and Firearm Suicide in the Absence and Presence of SYG Laws
eTable 7. Estimated Associations of SYG Laws With Homicide and Firearm Homicide Rates Across the US Using Nonpenalized GLMMs With Polynomials
eTable 8. Estimated Association of SYG Laws With Homicide and Firearm Homicide Rates by Race Across the US Using Data With Suspected Errors
eReferences

This supplemental material has been provided by the authors to give readers additional information about their work.

© 2022 Degli Esposti M et al. *JAMA Network Open.*

eMethods. Stand Your Ground and Self-Defense Laws by State

Stand Your Ground Laws

SYG laws expand historic legal principles governing where, and under what circumstances, an individual can use deadly force in self-defense. Specifically, the laws strengthen the legal right to use deadly force in public situations in which an imminent and immediate threat is reasonably perceived – even when safe retreat is possible – often granting both criminal and civil immunity.[1] The laws overwrite the common law principle of "duty to retreat", which stipulates that the use of lethal violence is only justified after attempting to avoid or retreat from a perceived threat.[2] An exception is the so-called "Castle Doctrine", where retreat is not required in situations where individuals encounter threats within the home (e.g., home invasions) because of the old English axiom "a man's home is his castle", representing the last place one can retreat to. SYG laws can therefore be seen an extension of the 'no duty to retreat' principle embedded within the Castle Doctrine to any public setting where an individual has a legal right to be.[3] In addition, SYG laws have introduced a lower standard of proof for the justified use of deadly force. They replace the objective legal test – which typically judged whether a "reasonable prudent person" would have acted the same in similar circumstance – with a reasonable "presumption of fear".[4] This change bypasses legal consideration (by prosecutors and the jury) of whether the circumstances warranted reasonable use of lethal force as it *presumes* reasonableness.[5]

We defined Stand Your Ground (SYG) laws as legislative statutes which extend the legal right to use lethal force in self-defense to anywhere the individual as the right to be (see Category 5 in Box S1). We do not include extensions to the Castle Doctrine for the use of lethal self-defense to the person's place of residence, work, or vehicle; nor do we include states that have upheld components of SYG laws by practice via case law (e.g., Colorado) or jury instructions (e.g., California). We adopted this definition since we hypothesized that expanding the legal right to use deadly force in public places is the primary driver of any changes to homicide. We further defined SYG states as US states that enacted these statutes between January 2000 and January 2016 to adequately model trends before and after SYG laws were enacted.

It is therefore important to note that this study does not evaluate the role of criminal (prosecutorial) or civil immunity in some SYG laws, or the impact of upholding SYG principles in practice, since the theoretical mechanism and timing of these more subtle legal changes are unclear. We also do not evaluate the impacts of SYG laws in seven states that enacted their SYG laws outside our defined intervention period: Utah (02/03/1994), Idaho (01/07/2018), Wyoming (01/07/2018), Ohio (06/04/2021), North Dakota (19/04/2021), and Arkansas (27/04/2021).

Self-Defense Laws by State

We used systematic methods to map out and categorize all 50 US states by their variant of self-defense law. District of Columbia was excluded from our analyses because it is a district not a state. First, we individually identified and investigated each states' legislation on self-defense. Second, we identified five categories and definitions of self-defense laws, described in Box S1. Third, we used pre-existing resources and databases,[6–10] as well as research,[11–16] to identify dates of enactment and classify states by their self-defense laws (eFigure 1). The details and timings of self-defense laws in each state is provided in eTable 1. Fourth, we applied our study period to identify SYG states (n=23), non-SYG states (n=18), and states that needed to be excluded from the analyses (n=9) due to potential contamination effects eTable 2.

© 2022 Degli Esposti M et al. *JAMA Network Open.*

eBox 1: Legal definitions of categories of self-defense laws

1) ***Duty-to-Retreat***
   Legal requirement that a person must retreat when threatened, if it is safe to do so, before using lethal force in self-defense
2) ***Castle Doctrine***
   Legal requirement that a person must retreat when threatened, if it is safe to do so, before using lethal force in self-defense unless the person is in their place of residence. If they are in their place of residence, then there is no duty-to-retreat and lethal force may be used if reasonable threat is perceived
3) ***Castle Doctrine – Expanded***
   Legal requirement that a person must retreat when threatened, if it is safe to do so, before using lethal force in self-defense unless the person is in their place of residence and/or place of work and/or vehicle. If they are in these places, then there is no duty-to-retreat and lethal force may be used if reasonable threat is perceived
4) ***Stand Your Ground law – In practice***
   Case law or jury instructions specify that there is not necessarily a legal requirement that a person must retreat when threatened, even if it is safe to do so, so long as the person is somewhere where they have a right to be and are not engaged in criminal activity
5) ***Stand Your Ground law – By statute***
   There is a legal right to use lethal force in self-defense where reasonable imminent threat is perceived, so long as the person is somewhere where they have a right to be and are not engaged in criminal activity

© 2022 Degli Esposti M et al. *JAMA Network Open.*

Outcomes and Data Cleaning

We restricted the study time period from January 1999 to December 2017 to obtain consistent ICD-10 codes over time for underlying cause of death – as prior to 1999 ICD-9 codes were used instead. Specifically, we used 113 selected causes of death groups for the ICD-10 codes, which are provided by the CDC to support research and analysis. eTable 3 specifies all four outcomes and their corresponding selected cause of death groups and ICD-10 names and codes.

All recording changes over time were investigated and harmonized (e.g., the introduction of categories *U01-*U03 for coding deaths due to acts of terrorism), and data extraction was validated through external checks and data visualizations. As part of these data visualizations, we systematically identified and excluded any outliers from one-off atypical events which resulted in mass death and removed these from the analyses. For example, the 9/11 terrorist attack on the twin towers. A full list of data outliers, which were identified and coded as missing, are specified in eTable 4.

Data checks also identified a potential error with the coding of homicide and firearm homicide by race in Texas from Sept 2007 to Feb 2009. Data checks included thorough data visualisations, as well as comparisons across data sources (e.g., the FBI's Supplementary Homicide Reports, see eFigure 2). We escalated this query to Texa's DSHS Center for Health Statistics, Vital Events Data Management team but, due to the COVID-19 pandemic, they did not have the capacity to investigate the issue (response received: 22/01/2021). We therefore replaced these data points by random sample imputation with bounded lower and upper values using *imputeTS*, see eFigure 3.[17] We present the imputed results in the main paper and the results using the original data with the suspected data error in eTables 6 & 7. Note, there were no identified errors in the overall counts of homicide and firearm homicide.

Model Equation

The underlying model for our main analyses can be represented by the following simplified equation:

$$log(Y_t) = log(n_t) + \alpha_s + f_s(t, \gamma) + h(m_t; \delta, q) + \beta x_t$$

where t is the sequence of times of observations, m the variable indicating the month (1 as January, 2 as February, and so on until 12 as December), and s is state. Y_t represents monthly counts (ie the outcome following a quasi-Poisson distribution) and log (n_t) represents the offset of the logarithm of the population from which the events originated; results are thus interpreted as monthly rates. The term α_s is a state-specific intercept and f_s is a smooth function describing state-specific long-term trends, while h models seasonality using harmonic terms – Fourier series of q pairs of sine and cosine terms with coefficients δ, which we consistently modelled as 3 and 12, respectively.[18] The variable x is a dummy variable indicating the presence of the law; taking the value of 1 in the presence of SYG law (in the post-law period in SYG states) and 0 in the absence of SYG law (non-SYG states and the pre-SYG law period in SYG states). The parameter β can be interpreted as the log-relative risk of the outcome – homicide or firearm homicide – in the presence vs. absence of the SYG law.

© 2022 Degli Esposti M et al. *JAMA Network Open.*

eAppendix. Evidence of Nonlinear Trends, Nonpenalized Approach, and Restricted Linear Analyses

Evidence of Non-Linear Trends

In addition to data visualizations of long-trend trends at the state and national level, we evaluated assumptions of linearity by comparing different model specifications. We fitted a series of generalized linear mixed models (GLMMs) and evaluated their model fit with respect to likelihood ratio (LR) tests and Akaike information criterion (AIC) to determine whether state-specific and national long-term trends were most parsimoniously described by linear or non-linear trends.

We found that model fit improved when modelling long-term trends with higher polynomials for both state-specific and national trends – as lower AIC values represent more parsimonious models (see eTable 5). LR tests were also used to directly compare whether model fit significantly improved (p<0.01) when using higher polynomials to describe trends. These findings indicate that both state-specific and national long-trend trends for homicide and firearm homicide violate assumptions of linearity. Evidence for non-linearity of trends was further supported through model diagnostics by inspecting the distributions of autocorrelation for linear (e.g., GLMMs with linear trends) versus non-linear (e.g., GAMs) models (eFigure 4).

Non-Penalized Approach

Our main analyses use a GAM framework to estimate the impacts of SYG laws. However, due to the penalisation approach, there may be concerns relating to overfitting and a reduced confidence in the p-values[19]. To check the robustness of our penalized approach, we conducted complementary analyses using a non-penalized approach: generalized linear mixed models (GLMMs). We fitted a series of GLMMs specifying a cubic term for national and state-specific time trends (i.e., fixed and random effects, respectively) as higher order polynomial terms resulted in superior model fit (eTable 5). Due to convergence problems for some of the stratified models, cubic terms for state-specific trends could not always be fitted. In these incidences, state-specific trends were fitted using the next highest order polynomial, quadratic. This non-penalized approach replicated our main GAM models, providing even higher estimates of the impacts of SYG laws (see eTable 8). This provides evidence to support confidence in our estimated effects and corresponding p values, indicating that our penalization approach provides more conservative estimates than a non-penalized approach.

Restricted Linear Analyses

Another potential drawback of using models with non-linear trends is a loss of certainty when forecasting the estimated counterfactual – what would have happened in the absence of the intervention. This is due to the increased flexibility, thus variability, when using polynomials and/or splines to fit underlying trends and forecast predicted trends. We therefore conducted a supplementary analysis of SYG states; restricting the study period from 36 months (3 years) before and 36 months (3 years) SYG laws were enacted. We modelled simple ITS quasi-Poisson regression analyses with linear trends and pooled the effect estimates for each SYG state using fixed effects meta-analysis. These restricted analyses replicated our main results, finding an average 9% increase in homicide rates (IRR=1.09; 95%CI: 1.05-1.13; p<0.0001) and firearm homicide rates (IRR=1.09; 95%CI: 1.04-1.14; p<0.0001) following the enactment of SYG laws (eFigures 8 & 9). We also continued to find no impact of SYG laws on monthly rates of suicide (IRR=0.99; 95%CI: 0.97-1.02; p=0.4643) and firearm suicide (IRR=0.99; 95%CI: 0.96-1.02; p=0.4681).

© 2022 Degli Esposti M et al. *JAMA Network Open.*

eFigure 1. Visualization of the Multiple Baselines Using Staggering of the Enactment of SYG Laws and Multiple Locations Using 23 SYG States During the Study Period, 1999 to 2017

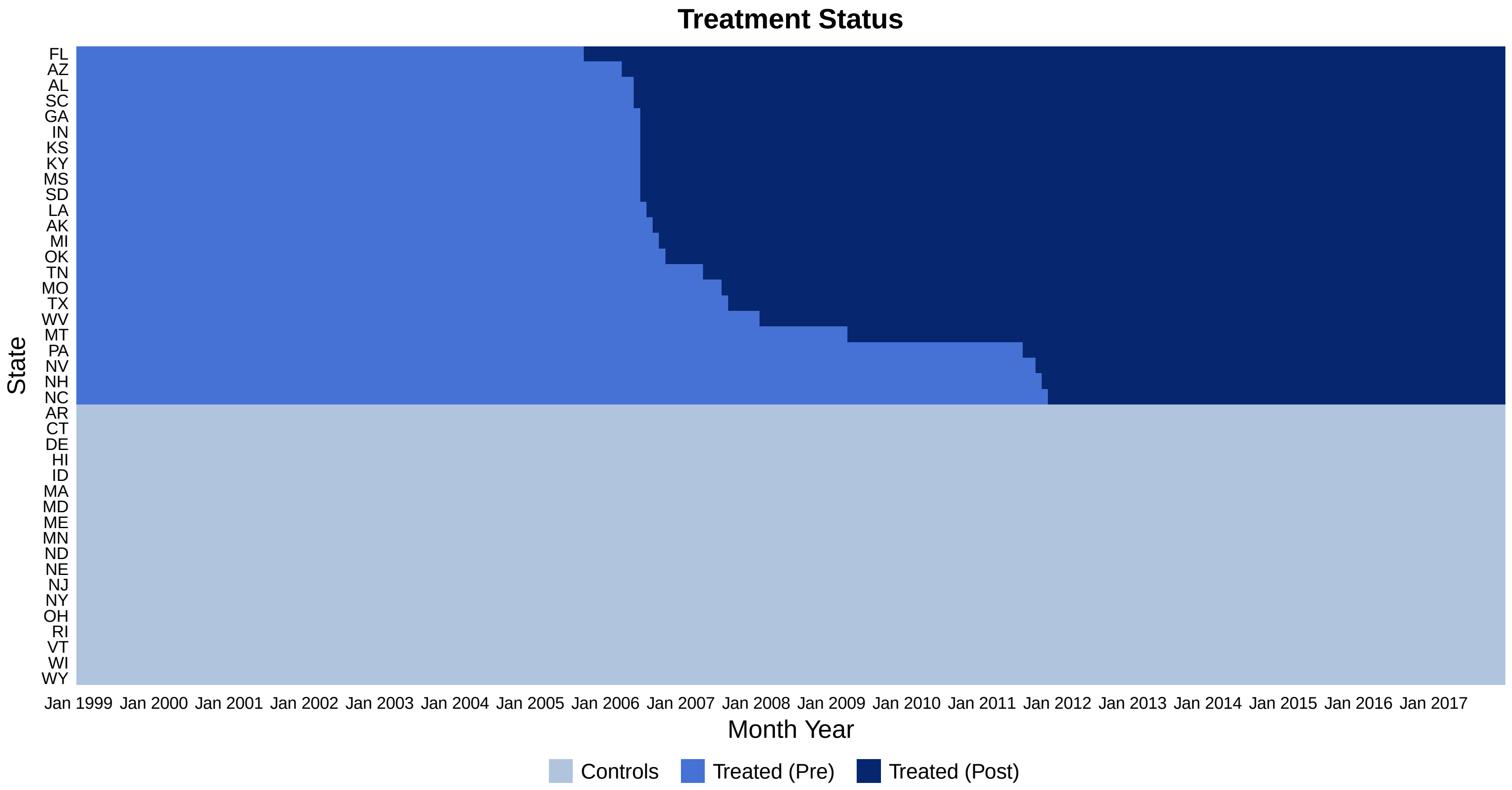

© 2022 Degli Esposti M et al. *JAMA Network Open.*

eFigure 2. Data Visualization Showing the Suspected Coding Error in the Data for Race in Texas From September 2007 to February 2009

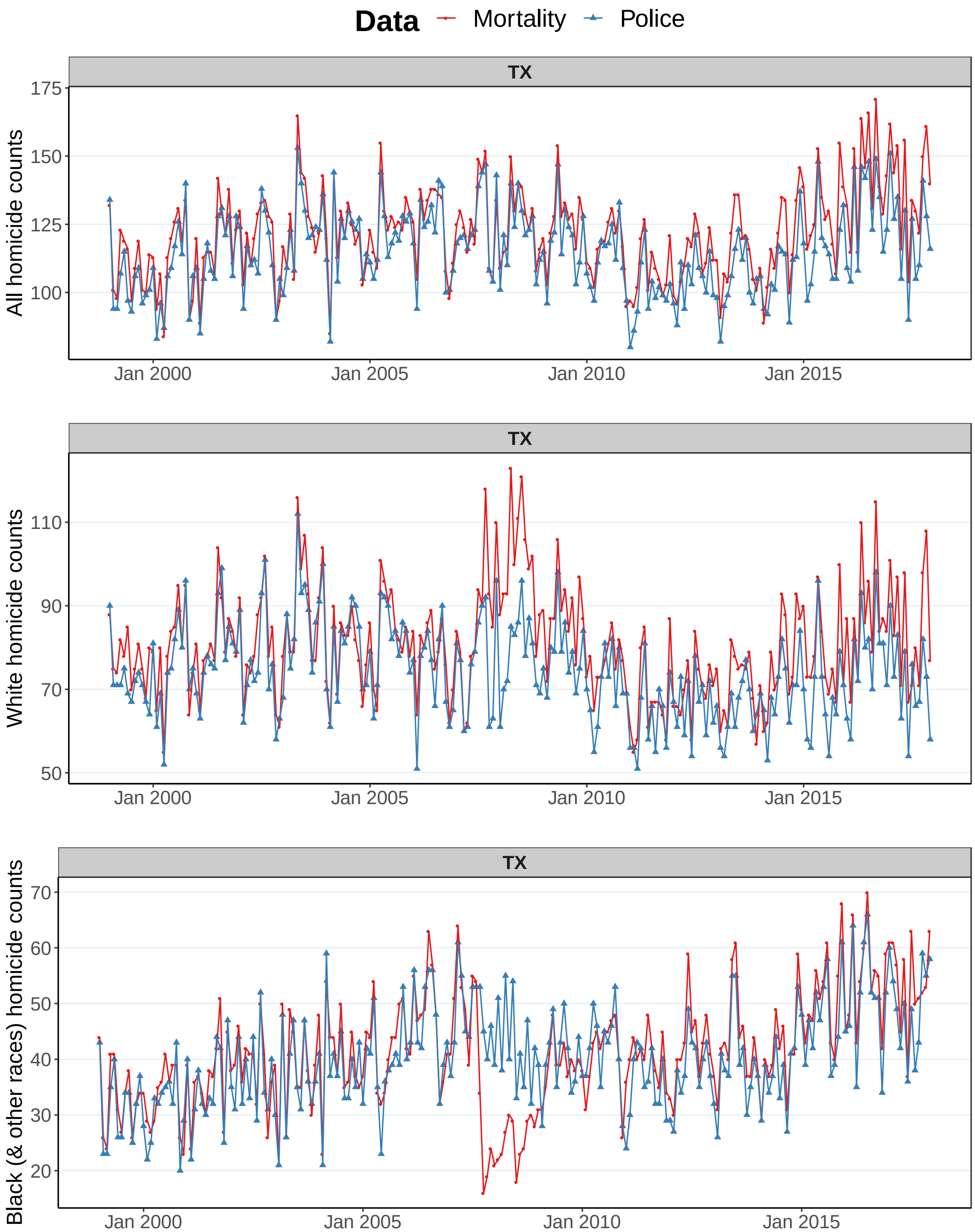

Mortality *(red)* are CDC's Restricted Use Vital Statistics on Multiple Cause of Death: the data source for this manuscript. Police *(blue)* are the FBI's Supplementary Homicide Reports: a second source included for data checking.

© 2022 Degli Esposti M et al. *JAMA Network Open.*

eFigure 3. Plots Showing Imputed Data by Bounded Random Sampling to Correct for the Suspected Coding Error in the Data for Race in Texas From September 2007 to February 2009

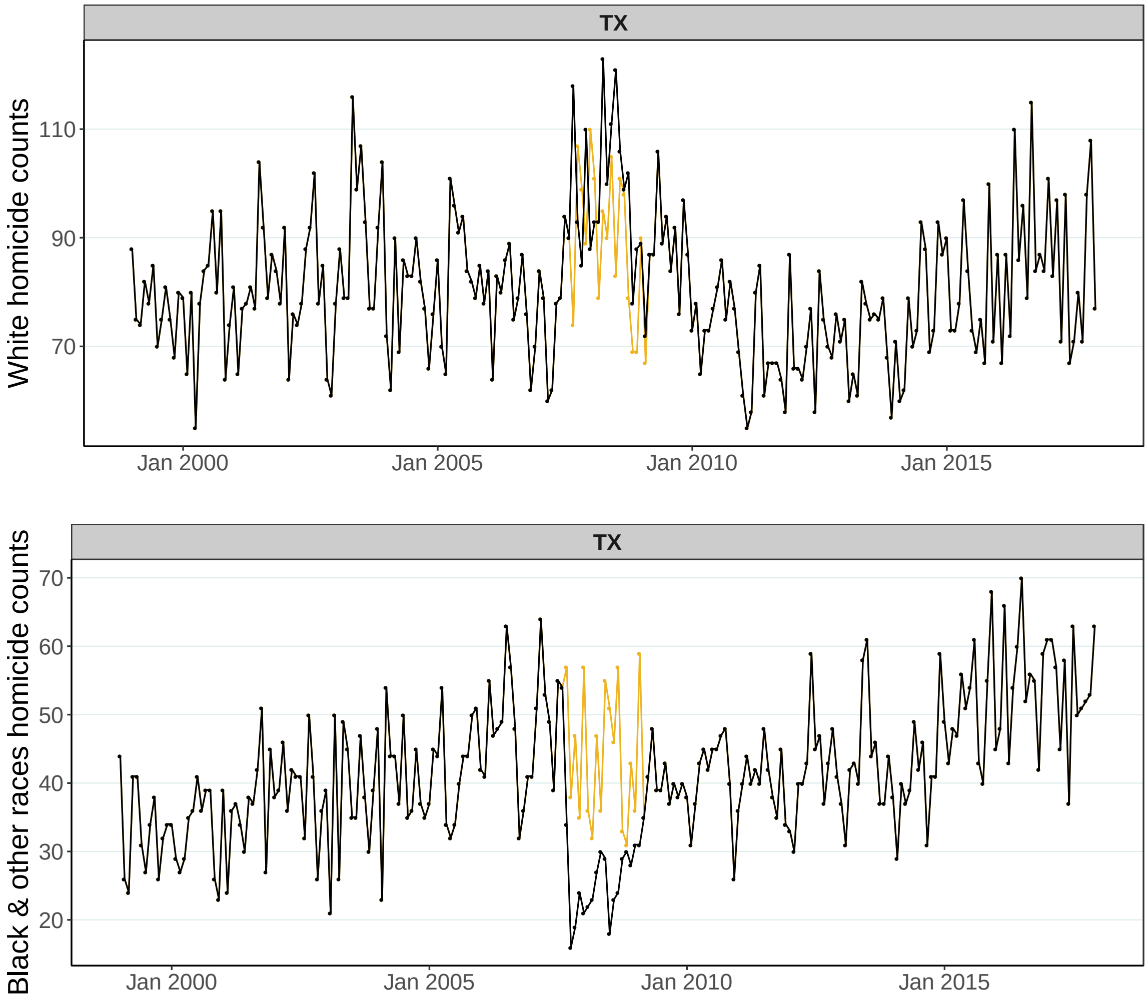

(Refer to eFigure 3). Imputed values are shown in yellow.

© 2022 Degli Esposti M et al. *JAMA Network Open.*

eFigure 4. Plots of Partial Autocorrelation Functions for GLMM With Linear Trends and GAMs for Homicide and Firearm Homicide

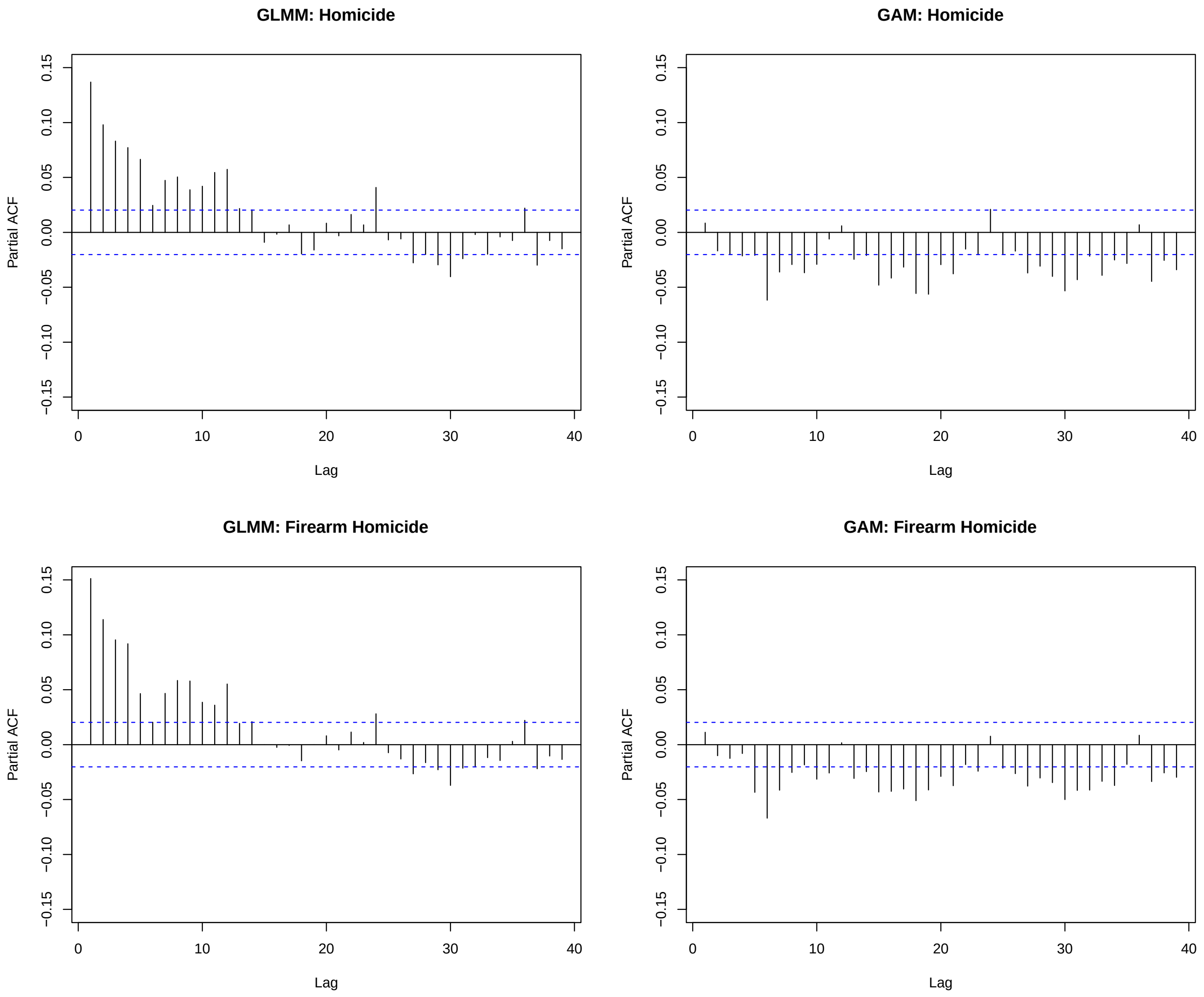

© 2022 Degli Esposti M et al. *JAMA Network Open.*

eFigure 5. Estimated Associations of SYG Laws With Monthly Homicide Rates Across the US

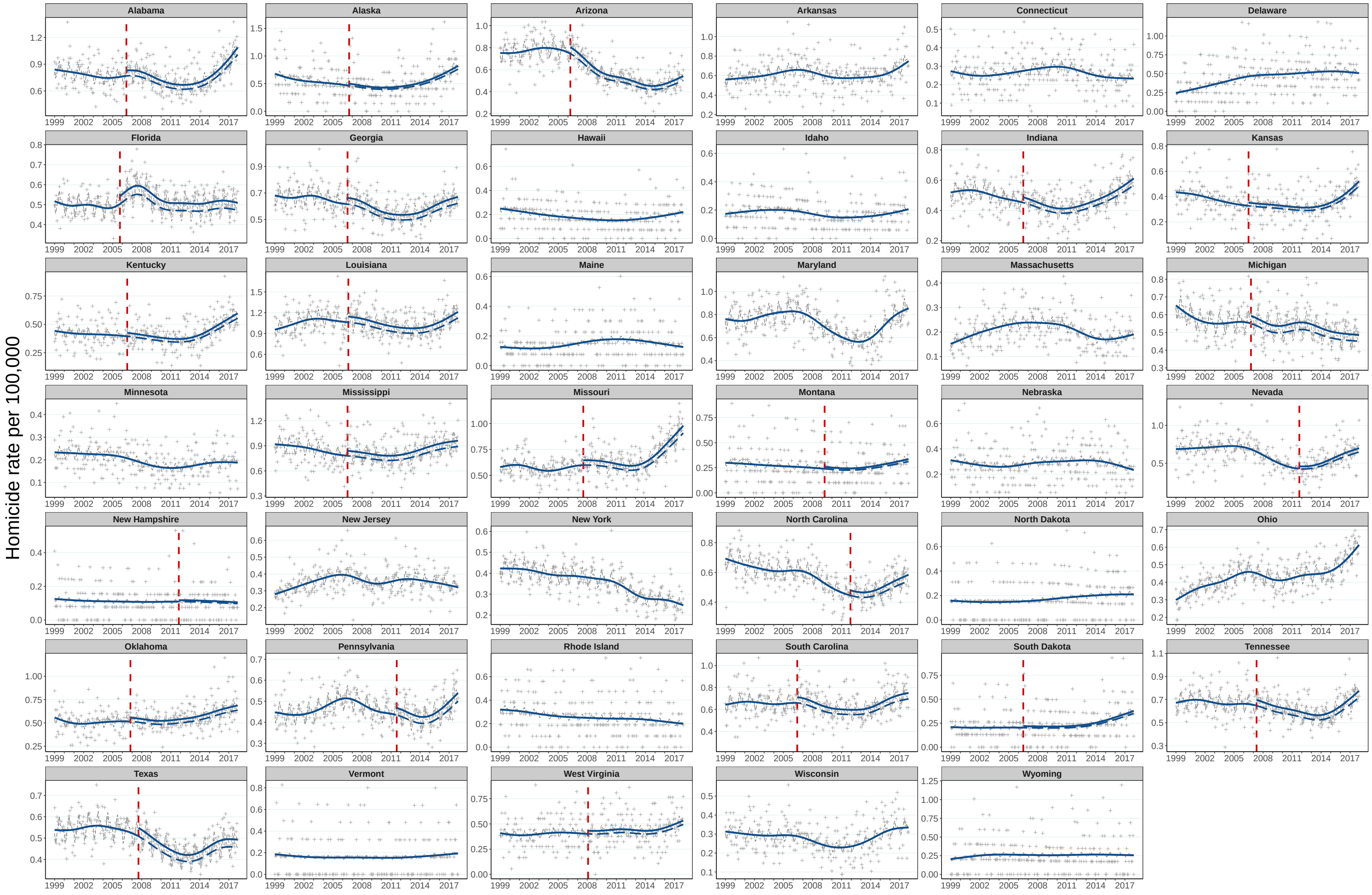

© 2022 Degli Esposti M et al. *JAMA Network Open.*

eFigure 6. Estimated Associations of SYG Laws With Monthly Firearm Homicide Rates Across the US

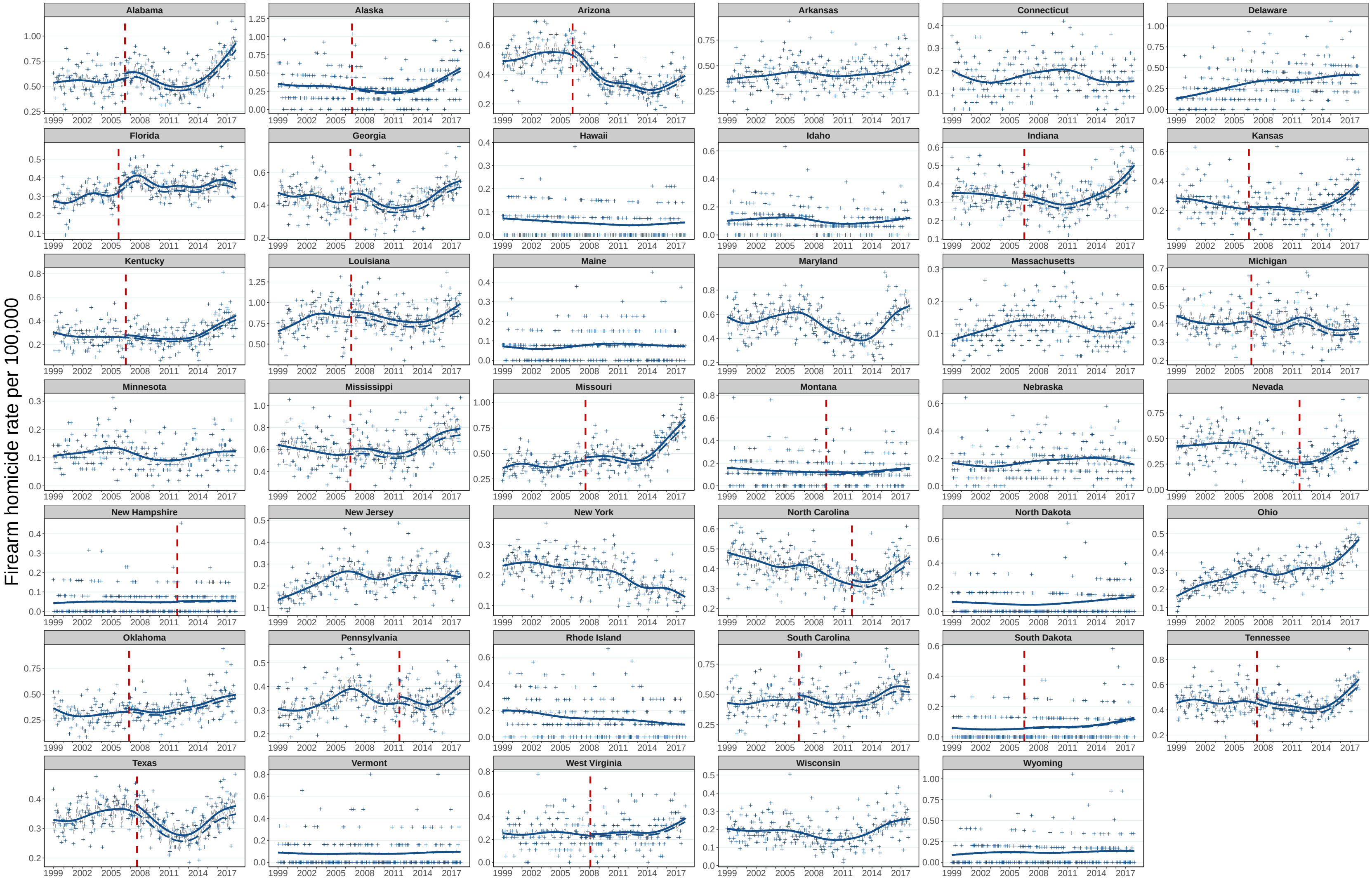

© 2022 Degli Esposti M et al. *JAMA Network Open.*

eFigure 7. Estimated Associations of SYG Laws With Monthly Suicide Rates Across the US

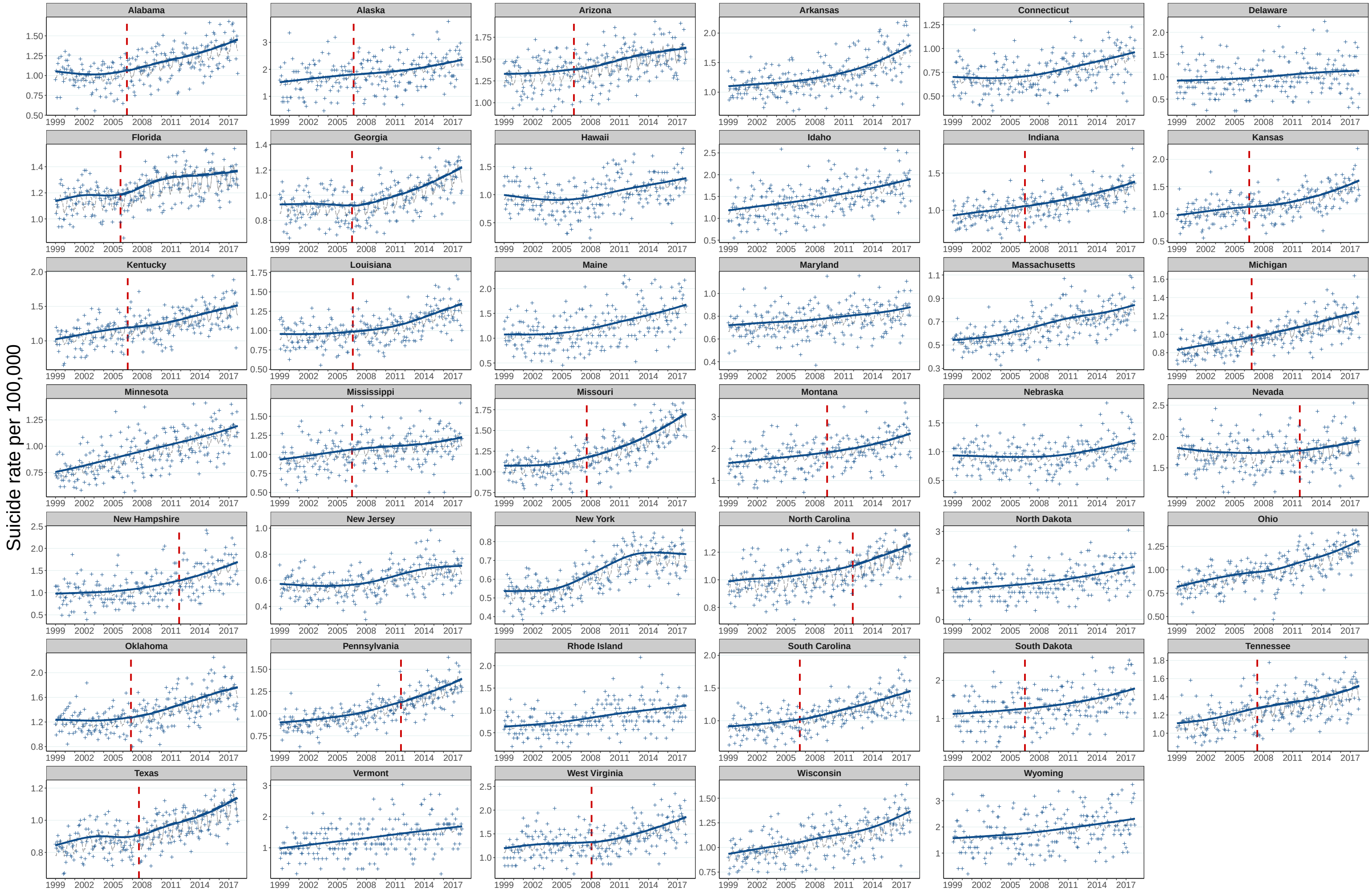

© 2022 Degli Esposti M et al. *JAMA Network Open.*

eFigure 8. Estimated Associations of SYG Laws With Monthly Firearm Suicide Rates Across the US

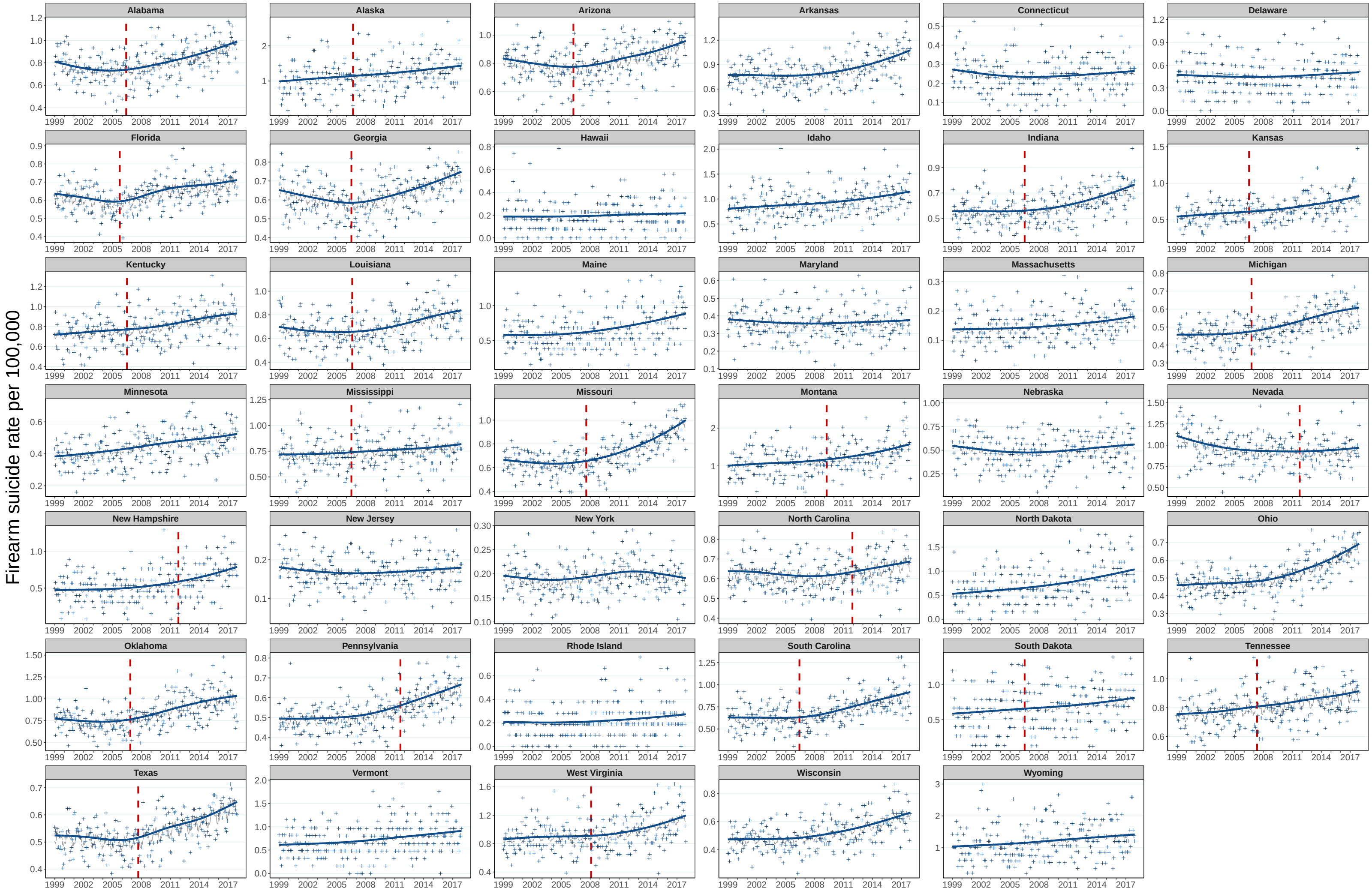

© 2022 Degli Esposti M et al. *JAMA Network Open.*

eFigure 9. Estimated Associations of SYG Laws With Monthly Homicide and Firearm Homicide Rates in SYG States by Restricted ITS Models With Linear Trends

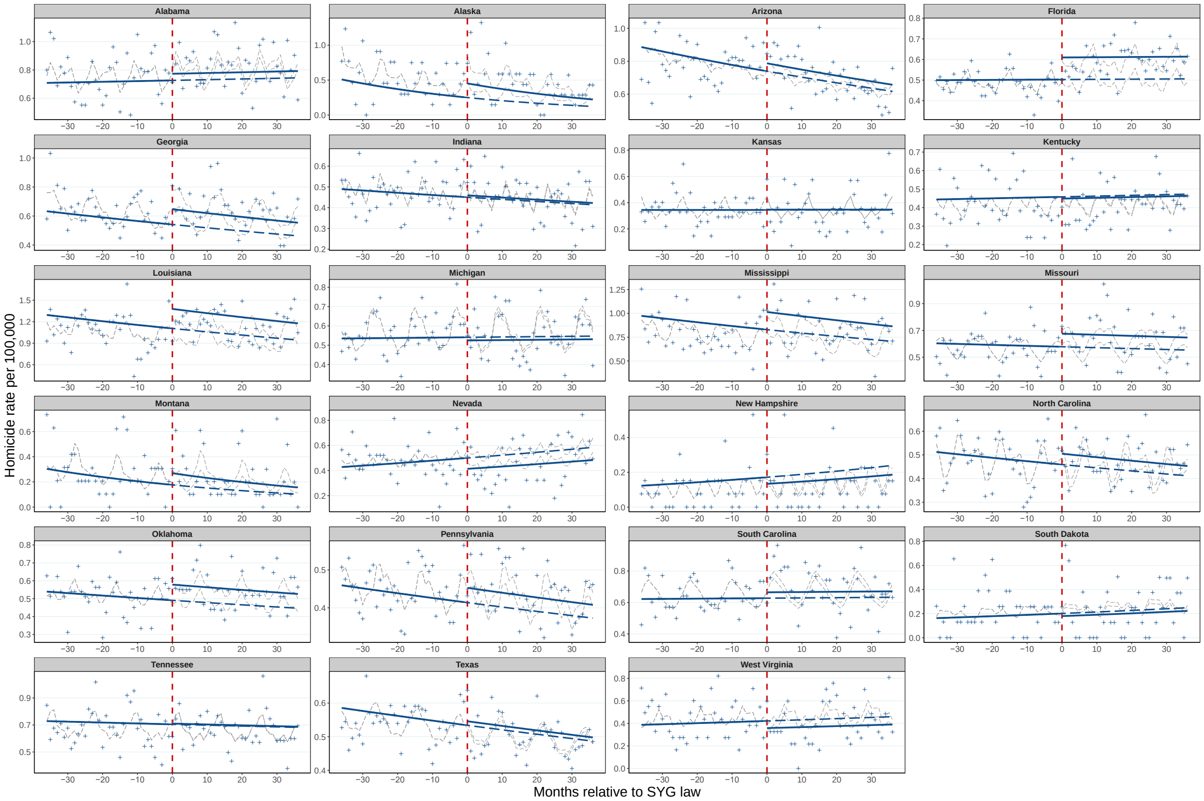

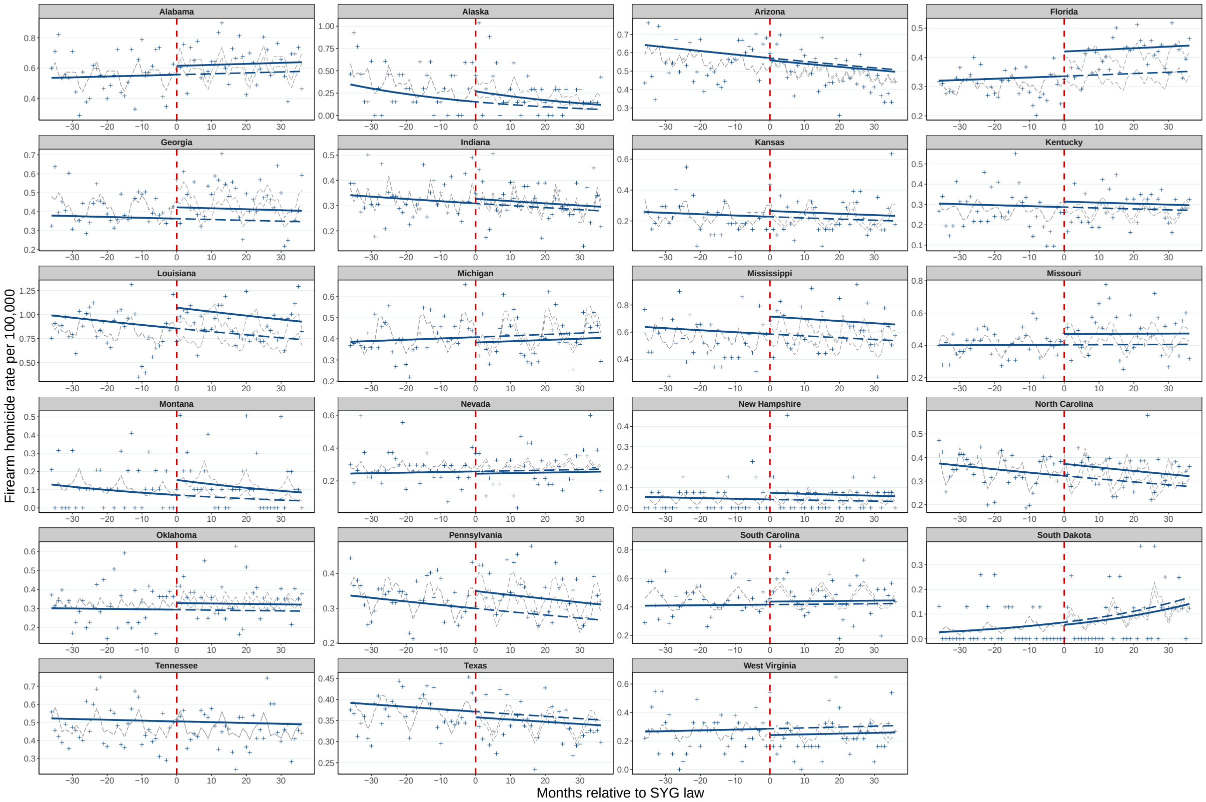

© 2022 Degli Esposti M et al. *JAMA Network Open.*

eFigure 10. Forest Plot of Fixed-Effects Meta-analysis Pooling Restricted ITS Models With Linear Trends for the Association of SYG Laws With Homicide and Firearm Homicide Rates in SYG States

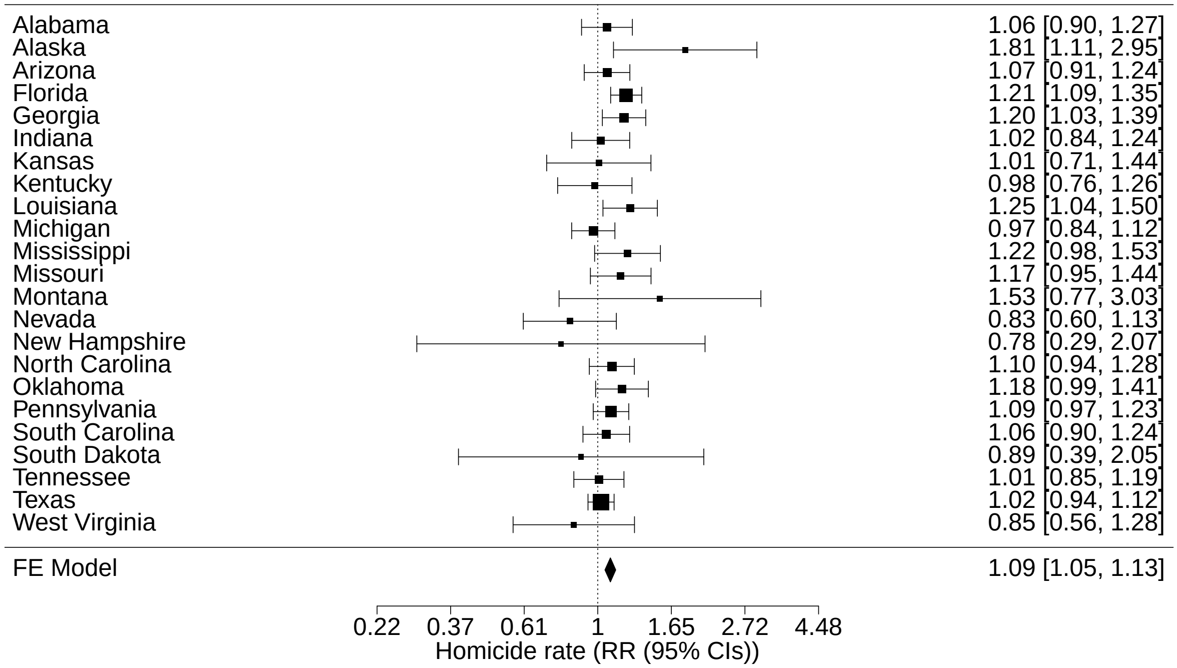

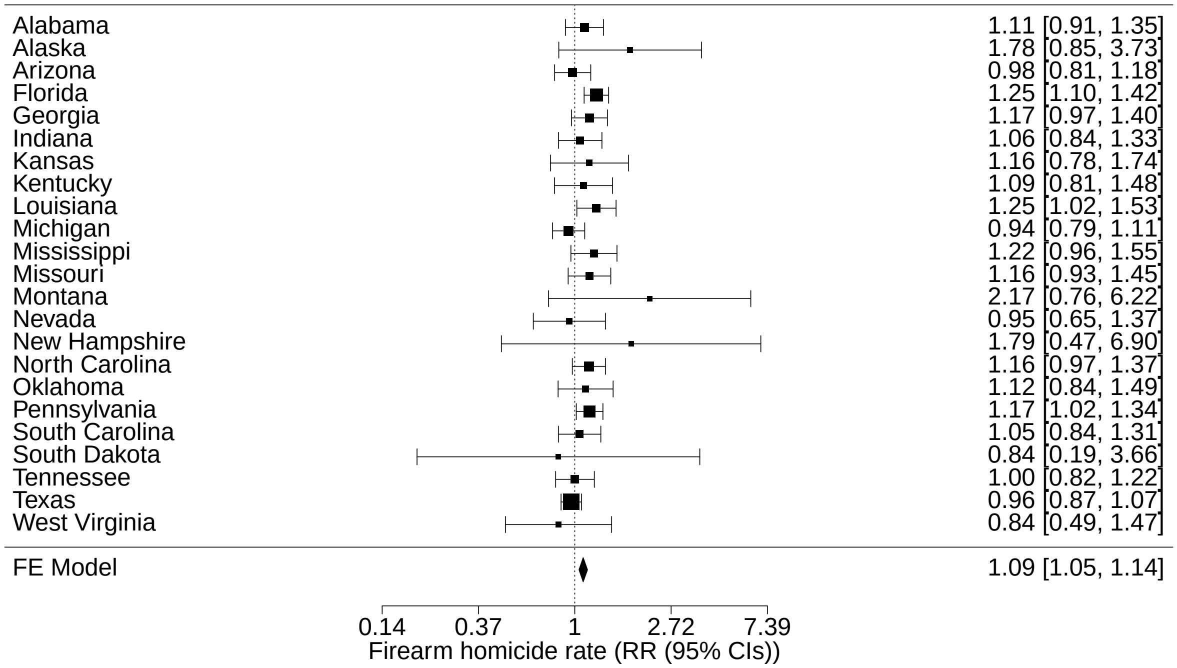

© 2022 Degli Esposti M et al. *JAMA Network Open.*

eFigure 11. State-Specific Associations of SYG Laws With Monthly Homicide Rates Estimated by Separate ITS Models With Nonlinear Trends for Each SYG State

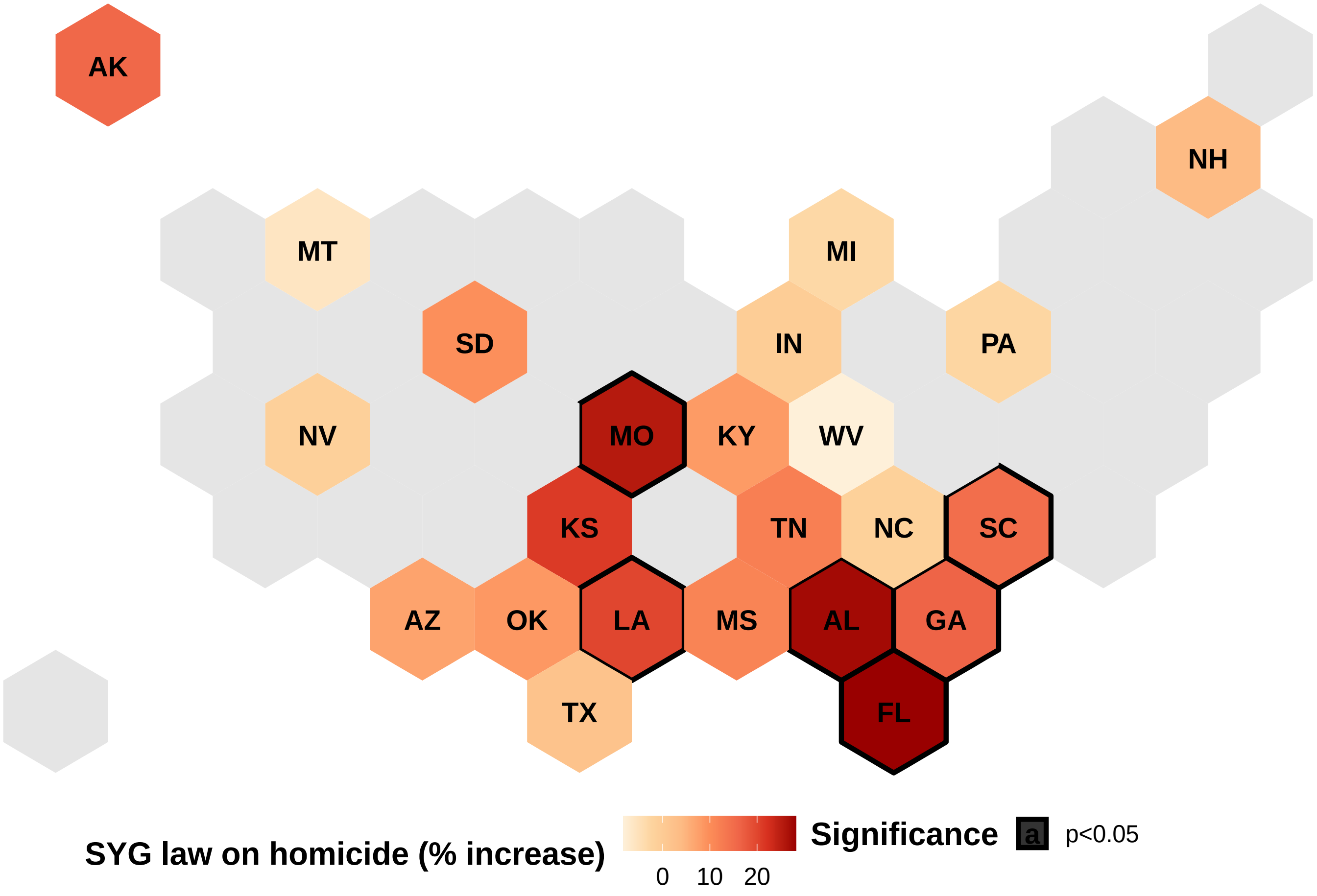

© 2022 Degli Esposti M et al. *JAMA Network Open.*

eTable 1. Self-defense Laws Across All 50 US States (Excluding District of Columbia)

State	Category of self-defense law	Details	Stand Your Ground laws	
			Statute Number	Date of enactment
Alabama	Stand Your Ground – By statute	..	13A-3-23	01/06/2006
Alaska	Stand Your Ground – By statute	..	11.81.335	13/09/2006
Arizona	Stand Your Ground – By statute	..	13-411	24/04/2006
Arkansas	Stand Your Ground – By statute	..	HB 1898	27/04/2021
California	Stand Your Ground – In practice	Jury instruction *California Penal Code Section 198.5; CALCRIM 505, 506*	..	..
Colorado	Stand Your Ground – In practice	Case law *Cassels v. People, 92 P.3d 951 (CO Supreme Court 2004)*	..	..
Connecticut	Castle Doctrine – Expanded	Place of work	C.C.J.I. 2.8-3, 53a-19	01/07/2006
Delaware	Castle Doctrine – Expanded	Place of work	464, 465	01/07/2017
Florida	Stand Your Ground – By statute	..	776.012, 776.013, 776.031	01/10/2005
Georgia	Stand Your Ground – By statute	..	16-3-23.1, 16-3-21	01/07/2006
Hawaii	Castle Doctrine – Expanded	Place of work	..	..
Idaho	Stand Your Ground – By statute		19-202A	01/07/2018
Illinois	Stand Your Ground – In practice	Case law *In Re T.W., 888 N.E.2d 148 (IL Ct. App. 2008); IPJI-Crim 24-25.09X*	..	..
Indiana	Stand Your Ground – By statute	..	35-41-3-2	01/07/2006
Iowa	Stand Your Ground – By statute	..	704.1	01/07/2017
Kansas	Stand Your Ground – By statute	..	21-5222, 21-5223, 21-5230	01/07/2006
Kentucky	Stand Your Ground – By statute	..	503.050, 503.055	12/07/2006
Louisiana	Stand Your Ground – By statute	..	14:20	15/08/2006
Maine	Castle Doctrine	..	.	.
Maryland	Castle Doctrine	..	.	.
Massachusetts	Castle Doctrine	..	.	.
Michigan	Stand Your Ground – By statute	..	780.972	01/10/2006
Minnesota	Castle Doctrine	..	.	.
Mississippi	Stand Your Ground – By statute	..	97-3-15	01/07/2006
Missouri	Stand Your Ground – By statute	..	563.031	28/08/2007
Montana	Stand Your Ground – By statute	..	45-3-102, 45-3-103, 45-3-104, 45-3-110	27/04/2009
Nebraska	Castle Doctrine – Expanded	Place of work	.	.
Nevada	Stand Your Ground – By statute	..	200.120	01/10/2011

© 2022 Degli Esposti M et al. *JAMA Network Open.*

State	Category of self-defense law	Details	Stand Your Ground laws	
			Statute Number	Date of enactment
New Hampshire	Stand Your Ground – By statute	..	627:4, 627:7	11/11/2011
New Jersey	Castle Doctrine	..	..	..
New Mexico	Stand Your Ground – In practice	Jury instruction *UJI 14-5190*	..	..
New York	Castle Doctrine	..	..	..
North Carolina	Stand Your Ground – By statute	..	14-51.3, 14-51.2	01/12/2011
North Dakota	Stand Your Ground – By statute	..	HB 1498	19/04/2021
Ohio	Stand Your Ground – By statute	..	2307.601, 2901.05, 2901.09	06/04/2021
Oklahoma	Stand Your Ground – By statute	..	1289.25	01/11/2006
Oregon	Stand Your Ground – In practice	Case law *State v. Sandoval, 156 P.3d 60 (OR Supreme Court 2007)*	..	..
Pennsylvania	Stand Your Ground – By statute	..	505(b)(2.3)	29/08/2011
Rhode Island	Castle Doctrine	..	.	.
South Carolina	Stand Your Ground – By statute	..	16-11-420, 16-11-440, 16-1-60	09/06/2006
South Dakota	Stand Your Ground – By statute	..	22-18-14	01/07/2006
Tennessee	Stand Your Ground – By statute	..	39-11-611	22/05/2007
Texas	Stand Your Ground – By statute	..	9.31, 9.32	01/09/2007
Utah	Stand Your Ground – By statute	..	76-2-402	02/03/1994
Vermont	Duty-to-Retreat	..	..	..
Virginia	Stand Your Ground – In practice	Case law *Event v. Commonwealth 688 S.E.2d 244 (VA Supreme Court 2010)*	..	..
Washington	Stand Your Ground – In practice	Jury instruction *WCJI/WPIC 16.08, 17.05*	..	..
West Virginia	Stand Your Ground – By statute	..	55-7-22	28/02/2008
Wisconsin	Castle Doctrine – Expanded	Place of work & vehicle	..	..
Wyoming	Stand Your Ground – By statute	..	6-2-602	01/07/2018

Existing databases and resources,[6–10] and research,[11–16] were used to systematically classify all 50 US states by their variant of self-defence law.

© 2022 Degli Esposti M et al. *JAMA Network Open.*

eTable 2. Intervention SYG States, Comparison Non-SYG States, and Excluded States

Definitions for study evaluation	Number of states	States
Intervention SYG states[a]	23	Alabama, Alaska, Arizona, Florida, Georgia, Indiana, Kansas, Kentucky, Louisiana, Michigan, Mississippi, Missouri, Montana, Nevada, New Hampshire, North Carolina, Oklahoma, Pennsylvania, South Carolina, South Dakota, Tennessee, Texas, West Virginia
Comparison non-SYG states[b,c]	18	Arkansas, Connecticut, Delaware, Hawaii, Idaho, Maine, Maryland, Massachusetts, Minnesota, Nebraska, New Jersey, New York, North Dakota, Ohio, Rhode Island, Vermont, Wisconsin, Wyoming
Excluded states[c,d]	9	California, Colorado, Illinois, Iowa, New Mexico, Oregon, Utah, Virginia, Washington

[a] In total, seven states with SYG laws by statute were excluded from intervention states. Utah (02/03/1994), Idaho (01/07/2018), Wyoming (01/07/2018), Ohio (06/04/2021), North Dakota (19/04/2021), and Arkansas (27/04/2021) were excluded from this category as they enacted their SYG statutes outside the study period. Iowa (01/07/2017) was excluded because there was insufficient power to model post-intervention trends. [b] Idaho (01/07/2018), Wyoming (01/07/2018), Ohio (06/04/2021), North Dakota (19/04/2021), and Arkansas (27/04/2021) were included in comparison states since they did not enact or implement a SYG law during the study period (1999 to 2017). [c] Seven states that have upheld principles of SYG law by case law (Colorado, Illinois, Oregon, Virginia) and by jury instructions (California, New Mexico, Washington) were excluded from comparison states due to potential intervention cross-over effects. [d] Utah and Iowa were excluded from the evaluation since they had SYG laws enacted during the study period but there was no time-points to model pre-intervention trends for Utah and insufficient time-points to model post-intervention trends for Iowa. District of Columbia is not included in this study.

© 2022 Degli Esposti M et al. *JAMA Network Open.*

eTable 3. Outcomes and Their Corresponding Selected Cause of Death Group and *ICD-10* Details

Outcome	Outcome type	Cause of death group	ICD-10 name	ICD-10 codes
Homicide	Primary	127	Assault (homicide)	*U01.0-*U01.-*U01.9, *U02, X85-Y09, Y87.1
Firearm homicide	Secondary	128	Assault (homicide) by discharge of firearms	*U01.4, X93-X95
Suicide	Primary negative control	124	Intentional self-harm (suicide)	U03, X60-X84, Y87.0
Firearm suicide	Secondary negative control	125	Intentional self-harm (suicide) by discharge of firearms	X72-X74

eTable 4. Data Outliers Caused by 1-Off Events

Outcome	Data outlier	Event identified	Outcomes recoded as missing
Homicide	Colorado: 04/1999	Columbine School Shooting	White, Black & other races, 0-19y, male, female
	Connecticut: 12/2012	Sandy Hook Elementary School shooting	White, Black & other races, 0-19y, 20-34y, 35y+, male, female
	Hawaii: 11/1999	Xerox murders	White, Black & other races, 20-34y, 35y+, male, female
	Nevada: 10/2017	Las Vegas mass shooting	White, Black & other races, 20-34y, 35y+, male, female
	New York and Philadelphia: 09/2001	Terrorist attack on the Twin Towers	White, Black & other races, 0-19y, 20-34y, 35y+, male, female
	Virginia: 09/2001	Terrorist attack on the Pentagon	White, Black & other races, 0-19y, 20-34y, 35y+, male, female
Firearm homicide	Colorado: 04/1999	Columbine School Shooting	White, Black & other races, 0-19y, male, female
	Connecticut: 12/2012	Sandy Hook Elementary School shooting	White, Black & other races, 0-19years, 20-34y, 35y+, male, female
	Hawaii: 11/1999	Xerox murders	Black & other races, 20-34y, 35y+, male, female
	Nevada: 10/2017	Las Vegas mass shooting	White, Black & other races, 20-34y, 35y+, male, female

These outliers from one-off mass death events were excluded from the analyses. No outliers were identified or removed for suicide and firearm suicide. See eMethods section on “Outcomes and Data Cleaning”.

© 2022 Degli Esposti M et al. *JAMA Network Open.*

eTable 5. AIC Values for Different Combinations of Polynomials for Modelling State and National Trends in GLMMs for Homicides

National trends (fixed effects)	State-specific trends (random effects)		
	**Linear**	**Quadratic**	**Cubic**
**Linear**	54207.71	53825.63	53244.16
**Quadratic**	54159.44	53822.15	53245.50
**Cubic**	53736.71	53394.24	53229.59

GLMMs fitted using a Poisson distribution to obtain unbiased AIC measures. Models fitted with cubic national and cubic state-specific trends showed the most parsimonious fit as it has the smallest AIC value. AIC = Akaike information criterion; GLMMs = generalized linear mixed models.

© 2022 Degli Esposti M et al. *JAMA Network Open.*

eTable 6. Monthly Counts of Homicide, Firearm Homicide, Suicide, and Firearm Suicide in the Absence and Presence of SYG Laws

SYG state/outcome	Monthly counts, Mean (SD)		
	Jan 1999 to Dec 2017	Before law	After law
**SYG states** (n=23)			
Outcomes			
Homicide	35.20 (30.55)	33.51 (29.27)	36.63 (31.53)
Race: White[1]	16.45 (17.73)	16.38 (17.49)	16.51 (17.93)
Race: White[2]	16.49 (17.90)	16.38 (17.49)	16.58 (18.25)
Race: Black & other[1]	18.76 (16.41)	17.13 (15.35)	20.14 (17.13)
Race: Black & other[2]	18.71 (16.36)	17.13 (15.35)	20.05 (17.06)
Age: 0-19y	5.38 (5.32)	5.38 (5.45)	5.38 (5.21)
Age: 20-34y	15.16 (13.65)	14.42 (13.11)	15.78 (14.06)
Age: 35y+	14.60 (12.93)	13.63 (12.04)	15.42 (13.58)
Sex: Male	27.34 (24.02)	25.65 (22.78)	28.77 (24.94)
Sex: Female	7.86 (7.20)	7.86 (7.16)	7.85 (7.24)
Firearm homicide	24.76 (21.76)	22.62 (19.88)	26.58 (23.08)
Race: White[1]	10.12 (11.46)	9.79 (10.99)	10.40 (11.83)
Race: White[2]	10.15 (11.61)	9.79 (10.99)	10.46 (12.10)
Race: Black & other[1]	14.65 (13.43)	12.82 (12.04)	16.20 (14.33)
Race: Black & other[2]	14.61 (13.39)	12.82 (12.04)	16.12 (14.27)
Age: 0-19y	3.38 (3.58)	3.23 (3.49)	3.51 (3.65)
Age: 20-34y	12.40 (11.33)	11.46 (10.58)	13.20 (11.88)
Age: 35y+	8.95 (8.14)	7.89 (7.09)	9.85 (8.84)
Sex: Male	20.55 (18.29)	18.71 (16.75)	22.11 (19.37)
Sex: Female	4.21 (4.11)	3.91 (3.76)	4.48 (4.37)
Negative control outcomes			
Suicide	70.19 (59.61)	60.23 (48.74)	78.64 (66.32)
Firearm suicide	40.75 (32.39)	35.59 (26.95)	45.12 (35.81)
**Non-SYG states** (n=18)			
Outcomes			
Homicide	15.57 (20.01)	..	..
Race: White	6.31 (7.82)	..	..
Race: Black & other	9.26 (13.22)	..	..
Age: 0-19y	2.60 (3.67)	..	..
Age: 20-34y	7.10 (9.74)	..	..
Age: 35y+	5.85 (7.53)	..	..
Sex: Male	12.22 (16.18)	..	..
Sex: Female	3.35 (4.34)	..	..
Firearm homicide	9.95 (13.10)	..	..
Race: White	3.12 (4.00)	..	..
Race: Black & other	6.84 (10.00)	..	..
Age: 0-19y	1.50 (2.33)	..	..
Age: 20-34y	5.50 (7.66)	..	..
Age: 35y+	2.95 (4.00)	..	..
Sex: Male	8.58 (11.60)	..	..
Sex: Female	1.38 (1.98)	..	..
Negative control outcomes			
Suicide	36.62 (34.48)	..	..
Firearm suicide	15.61 (15.04)	..	..

Other races includes all races other than White or Black under the main categories of American Indian, Asian, and Pacific Islander.

[1] Values are based on imputed race data for Texas, Sept 2007–Feb 2009.

[2] Values are based on the original data which has suspected errors in its coding of race for Texas, Sept 2007–Feb 2009.

© 2022 Degli Esposti M et al. *JAMA Network Open.*

eTable 7. Estimated Associations of SYG Laws With Homicide and Firearm Homicide Rates Across the US Using Nonpenalized GLMMs With Polynomials

**Outcome**		**Step change, IRR (95% CI)**	**Polynomial modelling non-linear trends by state (i.e., random effects)**
Homicide		1.09 (1.07-1.12)***	Cubic
	Race: White	1.12 (1.05-1.16)***	Cubic
	Race: Black & other	1.08 (1.05-1.12)***	Quadratic
	Age: 0-19y	1.15 (1.09-1.22)***	Quadratic
	Age: 20-34y	1.09 (1.03-1.15)**	Cubic
	Age: 35y+	1.07 (1.04-1.11)**	Quadratic
	Sex: Male	1.09 (1.06-1.12)***	Quadratic
	Sex: Female	1.05 (1.01-1.10)**	Quadratic
Firearm homicide		1.09 (1.06-1.12)***	Quadratic
	Race: White	1.13 (1.08-1.17)***	Cubic
	Race: Black & other	1.09 (1.05-1.13)***	Quadratic
	Age: 0-19y	1.19 (1.11-1.28)***	Quadratic
	Age: 20-34y	1.10 (1.03-1.17)**	Cubic
	Age: 35y+	1.08 (1.03-1.13)***	Quadratic
	Sex: Male	1.09 (1.05-1.12)***	Quadratic
	Sex: Female	1.11 (1.04-1.17)***	Quadratic
Suicide		1.00 (0.98-1.01)	Cubic
Firearm suicide		1.00 (0.98-1.02)	Cubic

Incidence rate ratios by sociodemographic group are estimated by the stratified GLMMs with cubic terms specifying national (fixed effect) and state (random effect) trends. Quadratic terms were used to model state trends when models were unable to converge with the higher polynomial. Values based on imputed race data for Texas, Sept 2007–Feb 2009. Other races includes all races other than White or Black under the main categories of American Indian, Asian, and Pacific Islander. CI=confidence intervals; GLMMs= generalized linear mixed models; IRR= incidence rate ratios; SD=standard deviation. *p<0.05; **p<0.01; ***p<0.001.

© 2022 Degli Esposti M et al. *JAMA Network Open.*

eTable 8. Estimated Association of SYG Laws With Homicide and Firearm Homicide Rates by Race Across the US Using Data With Suspected Errors

**Outcome**		**Monthly rates per 100 000 population, Mean (SD)**				**Step change, IRR (95% CI)**	***P* value for significant differences between subgroups** (white *vs.* black & other races)
		**SYG states** (n=23)			**Non-SYG states** (n=18)		
		**Jan 1999 to Dec 2017**	**Before law**	**After law**	**Jan 1999 to Dec 2017**		
Homicide		0.55 (0.25)	0.54 (0.26)	0.55 (0.25)	0.31 (0.22)	1.08 (1.04-1.12)***	..
	Race: White	0.32 (0.17)	0.33 (0.18)	0.32 (0.16)	0.18 (0.14)	1.14 (1.08-1.19)***	<0.001
	Race: Black & other	1.45 (0.87)	1.45 (0.89)	1.46 (0.85)	0.96 (1.04)	1.00 (0.94-1.05)	
Firearm homicide		0.38 (0.21)	0.36 (0.20)	0.39 (0.22)	0.19 (0.17)	1.08 (1.03-1.13)***	..
	Race: White	0.20 (0.13)	0.20 (0.14)	0.20 (0.13)	0.09 (0.10)	1.16 (1.10-1.24)***	<0.001
	Race: Black & other	1.08 (0.77)	1.03 (0.76)	1.12 (0.79)	0.65 (0.78)	0.99 (0.92-1.05)	

Incidence rate ratios by sociodemographic group are estimated by the stratified models while *p* values are based on Wald tests comparing stratified models within each sociodemographic group. The results broken down by race are based on the original data, which has suspected errors in its coding of race for Texas from Sept 2007 to Feb 2009 (inclusive), for the imputed results see Table 1 in the manuscript. Other races includes all races other than White or Black under the main categories of American Indian, Asian, and Pacific Islander. CI=confidence intervals; IRR= incidence rate ratios; SD=standard deviation.

© 2022 Degli Esposti M et al. *JAMA Network Open.*

eReferences

1. Lave TR. Shoot to kill: A critical look at stand your ground laws. *University of Miami Law Review*. 2013;67:827-860.
2. Brown RM. *No Duty to Retreat: Violence and Values in American History and Society*. University of Oklahoma Press; 1994.
3. Hafetz JL. "A Man's Home is His Castle?": Reflections on the home, the family, and privacy during the late nineteenth and early twentieth centuries. *William & Mary Journal of Women and the Law*. 2002;8(2):175-242.
4. Jansen S, Nugent-Borakove EM. *Expansions to the Castle Doctrine: Implications for Policy and Practice*. National District Attorneys Association; 2016:1-24.
5. Ward CV. Three questions about "Stand Your Ground" laws. *Faculty Publications*. 2020;95(3):119-138.
6. Siegal M. State Firearms Laws Database, 1991-2020. State Firearm Laws. Published 2020. Accessed November 25, 2019. http://www.statefirearmlaws.org/
7. Cherney S, Morral AR, Schell TL, Smucker S. RAND State Firearm Law Database. Published 2019. Accessed October 16, 2019. https://www.rand.org/pubs/tools/TL283-1.html
8. American Bar Association. *National Task Force on Stand Your Ground Laws: Final Report and Recommendations*.; 2015:1-66. http://www.americanbar.org/content/dam/aba/images/diversity/SYG_Report_Book.pdf
9. Mayors Against Illegal Guns. *Shoot First: "Stand Your Ground" Laws and Their Effect on Violent Crime and the Criminal Justice System*. National Urban League; 2013.
10. Branca AF. *The Law of Self Defense: The Indispensable Guide for the Armed Citizen*. Law of Self Defense; 2013.
11. Wallace LN. Castle Doctrine Legislation: Unintended effects for gun ownership? *Justice Policy Journal*. 2014;11(2):1-17.
12. Munasib A, Kostandini G, Jordan JL. Impact of the Stand Your Ground law on gun deaths: Evidence of a rural urban dichotomy. *European Journal of Law and Economics*. 2018;45(3):527-554. doi:10.1007/s10657-018-9581-z
13. Cheng C, Hoekstra M. Does strengthening self-defense law deter crime or escalate violence? Evidence from expansions to Castle Doctrine. *Journal of Human Resources*. 2013;48(3):821-854.
14. Boots D, Bihari J, Elliott E. The State of the Castle: An overview of recent trends in state Castle Doctrine legislation and public policy. *Criminal Justice Review*. 2009;34(4):515-535. doi:10.1177/0734016809332095
15. Siegel M, Pahn M, Xuan Z, Fleegler E, Hemenway D. The impact of state firearm Laws on homicide and suicide deaths in the USA, 1991–2016: A panel study. *Journal of General Internal Medicine*. 2019;34(10):2021-2028.
16. McClellan C, Tekin E. Stand Your Ground Laws, homicides, and injuries. *J Human Resources*. 2017;52(3):621-653. doi:10.3368/jhr.52.3.0613-5723R2
17. Moritz S, Bartz-Beielstein T. imputeTS: Time series missing value imputation in R. *R J*. 2017;9(1):207.
18. Hunsberger S, Albert PS, Follmann DA, Suh E. Parametric and semiparametric approaches to testing for seasonal trend in serial count data. *Biostatistics*. 2002;3(2):289-298. doi:10.1093/biostatistics/3.2.289
19. Wood SN. *Generalized Additive Models: An Introduction with R*. Chapman and Hall/CRC; 2006.

© 2022 Degli Esposti M et al. *JAMA Network Open.*
